# Cerebral Organoids Repair Ischemic Stroke Brain Injury

**DOI:** 10.1007/s12975-019-00773-0

**Published:** 2019-12-30

**Authors:** Shu-Na Wang, Zhi Wang, Tian-Ying Xu, Ming-He Cheng, Wen-Lin Li, Chao-Yu Miao

**Affiliations:** 1 Department of Pharmacology, Second Military Medical University / Naval Medical University, 325 Guo He Road, Shanghai, 200433 China; 2 Department of Cell Biology, Second Military Medical University / Naval Medical University, 800 Xiang Yin Road, Shanghai, 200433 China

**Keywords:** Cerebral organoids, Brain damage repair, Functional recovery, Stroke, Transplantation

## Abstract

Stroke is the second leading cause of death and main cause of disability worldwide, but with few effective therapies. Although stem cell-based therapy has been proposed as an exciting regenerative medicine strategy for brain injury, there are limitations. The developed cerebral organoids (COs) represent a promising transplantation source for stroke that remains to be answered. Here, we transplanted COs at 55 days and explored the feasibility in the rat middle cerebral artery occlusion (MCAO) model of stroke. COs transplantation at 6 h or even 24 h after MCAO significantly reduces brain infarct volume and improves neurological motor function. Transplanted COs show the potential of multilineage differentiation to mimic in vivo cortical development, support motor cortex region-specific reconstruction, form neurotransmitter-related neurons, and achieve synaptic connection with host brain via in situ differentiation and cell replacement in stroke. Cells from transplanted COs show extensive migration into different brain regions along corpus callosum. The mechanisms underlying COs transplantation therapy are also associated with enhanced neurogenesis, synaptic reconstruction, axonal regeneration and angiogenesis, and decreased neural apoptosis with more survival neurons after stroke. Moreover, COs transplantation promotes predominantly exogenous neurogenesis in the transplantation periphery of ipsilateral cortex and predominantly endogenous neurogenesis in the hippocampus and subventricular zone. Together, we demonstrate the efficacy and underlying mechanisms of COs transplantation in stroke. This preliminary but promising study provides first-hand preclinical evidence for COs transplantation as a potential and effective intervention for stroke treatment.

**Electronic supplementary material:**

The online version of this article (10.1007/s12975-019-00773-0) contains supplementary material, which is available to authorized users.

## Introduction

Stroke is the second leading cause of death worldwide, endangering public health but with few effective therapies. Tissue plasminogen activator (tPA) is the only drug approved by FDA for the treatment of ischemic stroke and no drug is available for the treatment of hemorrhagic stroke [1, 2]. Although numerous neuroprotective agents have been studied in the past decades, they are unsuccessful or still in the preclinical-to-clinical transition [3]. With functional identification of stem cells, emerging researches have focused on regenerative medicine strategies using neural progenitor cell or neural stem cell (NPC/NSC) transplantation for stroke [4, 5]. The stem cell-based transplantation therapy offers protection for neural tissue in the acute phase of stroke, and achieves functional cell replacement of lost tissues via neurorestoration in the sub-acute and chronic phases of stroke [4, 5]. However, the cell source, limited cell survival, inadequate neuronal differentiation, and cell dose in stem cell transplantation are still challenges for translational application. Therefore, there is an urgent need for developing new effective therapies for stroke.

The recent generation of three-dimensional cerebral organoids (COs) from human pluripotent stem cells overcomes the limitations of stem cell-based transplantation therapy to a certain extent and shows advantages in diverse cell types (including but not limited to, NPCs, NSCs, mature and immature neurons, glia cells), rich cell source, considerable cell number, and controllable degree of cell differentiation and certain tissue volume (up to 4 mm in diameter) [6, 7]. Moreover, the COs show neural connectivity and brain functionality in vitro that recapitulates in vivo features of brain development and maturation [6, 8–10], providing possibility for directly repairing and replacing damaged brain tissue after stroke. Up to now, there are two reported studies about COs transplantation in the mouse cortex, both demonstrating the survival, vascularization, and neurodifferentiation of transplanted COs in the host brain [11, 12]. Compared to NPC transplantation, transplanted COs exhibit more cell survival number and better vascularization with host brain and harbor a large NSC pool with multilineage neurodifferentiation [12]. Therefore, COs represent a promising alternative source of NSCs to be a potential transplantation donor for stroke.

Considering no report of COs transplantation therapy in stroke, we plan to explore the efficacy of COs transplantation therapy, whether transplanted COs reduce brain damage volume and improve brain function recovery after stroke. The therapeutic time window for only drug t-PA is within 3 or 4.5 h after stroke onset according to current guidelines in different countries [13, 14]. Endovascular thrombectomy (EVT) is an accepted non-drug treatment for acute stroke with therapeutic time window of 6 h in the current guideline [14, 15]. Recently, the therapeutic time of EVT can be extended to 7.3 h with more stringent imaging selection criteria [16], and even to 24 h in carefully selected patients (DAWN trial) [17]. Therefore, the therapeutic time window of COs transplantation should be taken into consideration for its practical application in stroke. As such, we wonder whether it is feasible for COs transplantation at different therapeutic time window from 6 h to 24 h, or even 7 days after stroke onset.

Our group has performed serval studies on stroke and post-ischemia neurogenesis and angiogenesis [2, 18–20]. Therefore, this study is planned to transplant COs at 55 days and explore the feasibility, efficacy, and underlying mechanisms in stroke, hoping to provide first-hand preclinical evidence for COs transplantation as a potential and effective intervention for stroke.

## Materials and Methods

### Animals

All Sprague-Dawley rats (male, 250 ± 30 g) were purchased from Sino-British SIPPR/BK Lab Animal Ltd. (Shanghai, China). All rats received humane care, and all animal experiments were approved by the Institutional Animal Care and Use Committee of the Second Military Medical University, China. All rats were kept in a 12-h light/dark cycle with free access to food and water throughout the study. All animal studies in this article were in compliance with the ARRIVE guidelines [21].

### Human Embryonic Stem Cell Culture

H1 human embryonic stem cells (hESCs) were obtained from WiCell (Madison, WI, USA) and cultured as previously described with minor modifications [22]. In feeder-free mTesR™1 maintenance medium (STEMCELL, Canadian) and Matrigel (Corning, NY, USA, hESC-Qualified) coated plates, hESCs were passaged by using TrypLE (Gibco, MA, USA) at a ratio of 1:3 to 1:6 every 4–6 days. Then, hESCs were collected and seeded in the Matrigel coated 6-well tissue culture plates. Y-27632 ROCK inhibitor of 10 μM (Merck, Darmstadt, Germany) was indispensable to the first day of passage in mTesR™1 maintenance medium. All hESCs used in this study displayed optimal features of pluripotency with no evidence of differentiation. The use of hESCs in this study obeyed Ethical Guiding Principles for the Research of Human Embryonic Stem Cell.

### Generation of Cerebral Organoids

Cerebral organoids (COs) were generated as previously described with minor modifications [7]. hESCs at about 80% confluence were digested into single cells by Accutase (Gibco, MA, USA). A total volume of 150 μL containing 1.35 × 10^4^ cells was added into the each well of an ultra-attachment 96-well plate (Corning, NY, USA) with low bFGF medium containing DMEM/F12 (Invitrogen, MA, USA), 20% KoSR (Invitrogen, MA, USA), 3% hES-quality FBS (Gibco, MA, USA), 1% GlutaMAX (Invitrogen, MA, USA), 1% MEM-NEAA (Invitrogen, MA, USA), 55 μM 2-Mercaptoethanol (Merck, Darmstadt, Germany), 4 ng/ml bFGF (Peprotech, NJ, US), and 50 μM ROCK inhibitor Y-27632 (Merck, Darmstadt, Germany). The first process in ultra-attachment 96-well plate was to generate embryonic bodies (EBs) and maintained for 4–5 days. When the diameter of single EBs was about 350 μm, the culture medium was replaced with hES medium (Low bFGF medium without bFGF and Y-27632). When the diameter of EBs was about 500 μm with brightened smooth surface and quite dark center, EBs were transferred into ultra-attachment 24-well tissue culture plates. The culture medium was replaced with neural induction medium containing DMEM-F12 with 1% N2 supplement, 1% GlutaMAX supplement, 1% MEM-NEAA, and heparin (final concentration of 1 μg/ml) for 4–6 days to begin the induction of primitive neuroepithelia. Once the brighter outside of EBs showed radial organization of a pseudostratified epithelium consistent with neuroepithelium formation, the neuroepithelial tissues were transferred into Matrigel droplets with COs differentiation medium containing 50% DMEM/F12, 50% Neurobasal medium, 0.5% N2 supplement, 1% GlutaMAX supplement, 0.5% MEM-NEAA, 2.8 ng/ml human insulin solution (Sigma-Aldrich, St. Louis, MO, USA), 55 μM 2-Mercaptoethanol and 1% B27 supplement (without vitamin A). The Matrigel droplets were maintained for 4 days in a stationary condition with the formation of neuroepithelial buds, and then transferred into spinning bioreactor in Micro-Stir Slow Speed Magnetic Stirrers (Wheaton, Germany) at the speed of 85 rpm. COs were cultured with COs differentiation medium with 1% B27 supplement (with vitamin A) and changed every 4–7 days.

The COs used in this study were well qualified with typical identification in relevant culture process, namely brightened smooth surface of EBs, radial pseudostratified epithelium organization, and neuroepithelium formation before Matrigel droplets, outgrowths, and defined formation of neuroepithelial buds in Matrigel droplets. At the same time, the morphological characteristics were verified by microscope and the specific identities were confirmed by antibody specific immunofluorescence in different culture time of COs.

### Cerebral Organoids for Cryo-sectioning and Immunofluorescence Staining

COs were fixed in 4% (*w*/*v*) PFA for 15 min and dipped in 30% (*w/v*) sucrose solution at 4 °C overnight. Twelve hours later, warm gelatin/sucrose solution was used to embed COs. Cryo-sections of COs and immunofluorescence staining were performed under standard procedures. Antibodies used in this study are listed in Supplementary Table 1.

### Middle Cerebral Artery Occlusion Model

Middle cerebral artery occlusion (MCAO) surgery in rats was performed as described [23]. The rats were anesthetized with a combination of ketamine (50 mg/kg), xylazine (2.6 mg/kg), and acepromazine (0.50 mg/kg) by intraperitoneal injection. Briefly, rats underwent MCAO by electrocoagulation. All rats were given Cyclosporin A (10 mg/kg, i.p.) the day before transplantation surgery. Craniotomy was made along the midline of brain with a longitudinal incision of approximately 4 cm. The fascia of the skull was separated and a piece of skull over the left cortex was excised with 1.5 cm length and 0.6 cm breadth using a drill. In order to accommodate transplanted COs, we did transplantation surgery to make a cavity (3 mm in diameter, 2 mm in depth) in left motor cortex (center position: brain midline: 3.5 mm, bregma posterior: 0.5 mm) by biopsy punch. All rats were randomly grouped: Sham, MCAO, and COs transplantation groups. The rats in the Sham group were only performed craniotomy without brain injury. The rats in the MCAO group were performed with MCAO and transplantation surgery without COs transplantation. The rats in the transplantation group were performed with surgery of MCAO and COs transplantation (two COs at 55 days were implanted into the cavity at 6 h, 24 h, or 7 days after MCAO). Finally, the piece of excised skull and the bone wax were used together to seal the skull window. The incision was sutured and wiped with erythromycin ointment to prevent infection. All operations were performed under aseptic conditions. Cyclosporine A was intraperitoneally injected every other day until the rats were sacrificed. There is no animal death after transplantation surgery. There are animal deaths after MCAO surgery but with no difference among the groups.

### TTC Staining for Morphological Examination

As for the morphological examination, brain was removed under anesthesia and placed into − 20 °C for 30 min, sliced transversely into six sections from anterior to posterior extremity with same thickness, stained with 2% 2,3,5-triphenyl tetrazolium chloride (TTC; Sigma-Aldrich, St. Louis, MO, USA) for 30 min in the dark, and fixed in 4% paraformaldehyde for 1 h. The images of brain sections were captured by Micro Scan 6.3 image scanner system (BenQ, Tianjin, China). The quantitative analysis of infarct volume was analyzed by comparison with contralateral brain volume as following calculation formula: % *I* = 100 × (*V*_c_-*V*_L_) / *V*_c_ [% *I* = percent of infarcted gray matter (lying between the first and last section taken) that is infarcted; *V*_c_ = total volume of (normal) brain parenchyma in the contralateral control hemisphere; *V*_L_ = total volume of normal brain parenchyma in the ipsilateral lesioned hemisphere]. The quantitative analysis of ipsilateral brain volume was analyzed by comparison with contralateral brain volume as following calculation formula: % *I* = 100 × *V*_L_ / *V*_c_ [% *I* = percent of survival brain parenchyma in the ipsilateral hemisphere (lying between the first and last section taken); *V*_c_ = total volume of (normal) brain parenchyma in the contralateral control hemisphere; *V*_L_ = total volume of normal brain parenchyma in the ipsilateral lesioned hemisphere].

### Brain Tissue Collection and Immunofluorescence Staining

Rat brain tissues were harvested under anesthesia with perfusion of 4% paraformaldehyde (pH 7.4) as before [19]. All brain tissues were collected carefully without disruption of intact brain. Frozen coronal slices (8 μm thickness) of rat brain were prepared in the cryostat (CM3050S; Leica Microsystems, Bannockburn, IL, USA). TritonX100 and blocking serum of 0.2% (*w*/*v*) were successively added into brain sections with incubation of 15 min and 2 h, respectively. With incubation in the specific primary antibodies at 4 °C overnight (see Supplementary Table 1), corresponding secondary antibodies (Alexa 488/594-conjugated or Cy3-conjugated) for 2 h at room temperature without light, and nuclei staining with DAPI for 10 min, brain sections were stained with immunofluorescence. The immunofluorescence was examined under FLUOVIEW FV1000 confocal laser scanning microscope (Olympus, Japan) or Pannoramic MIDI automatic digital slide scanner (3D HISTECH, Hungary).

BrdU immunostaining was performed as described [19, 24]. BrdU (Sigma-Aldrich, St. Louis, MO) was injected every other day until death (50 mg/kg, IP, dissolved in saline).

### Histology Examination

Brain tissues in 4% paraformaldehyde were dehydrated with 30% sucrose in formalin (pH = 7.4), embedded in paraffin, and cut into coronal slices (8-μm thickness). The sections were deparaffinized and stained with hematoxylin and eosin (HE) and examined under a light microscope (Leica Microsystems, Berlin, Germany).

### Immunohistochemistry Staining

The paraffin-coated coronal brain slices were used immunohistochemistry (IHC) staining as previous report [25]. Briefly, the sections were deparaffinized and then retrieved antigen in citric acid buffer (PH 6.0). With blocking in 8% donkey serum for 2 h at room temperature, the sections were successively incubated with specific primary antibodies (see Supplementary Table 1) and HRP-conjugated secondary antibody. Then, the fresh chromogenic substrate DAB was used to visualize the staining. Images were obtained with a digital microscope (Leica Microsystems, Berlin, Germany).

### Nissl’s Staining

Briefly, the paraffin-coated coronal brain slices were stained with 1% toluidine blue for 40 min at 60 °C. Then, the slices were dehydrated in 70%, 80% and 95% and 100% ethanol, respectively, and hyalinized with xylene. Images were obtained with a digital microscope (Leica Microsystems, Berlin, Germany).

### Behavior Tests

To evaluate behavioral dysfunction, modified neurological severity scores (mNSS) and beam walking test were used to assess the motor function of the rat with or without COs transplantation [26]. All rats were blinded to tester in all behavior studies, and the baseline of neurological motor function in all rats was the same, for example, rats for beaming walking test were trained for 1 week to ensure all of them can smoothly through the balance beam before MCAO surgery. Rats without changes of motor function at 24 h after MCAO surgery were excluded.

### mNSS Evaluation

The modified neurological severity scores (mNSS) were used in this study. The evaluation indexes as follows: forelimb flexion (0 score, none; 0.5 score, slightly flexion; 1.0 score, the shoulder flexion can surround the entire the forelimb flexion); twist (0 score, none; 0.5 score, slightly twist; 1.0 score, forelimbs and heads can reach the hind limbs); side push (0 score, equal on both sides; 0.5 score, the ipsilateral weakened; 1.0 score, the ipsilateral has no resistance); circle (0 score, none; 0.5 score, large circle; 1.0 score, small circle); hind limb placement (0 score, rapid recovery; 0.5 score, recovery delay; 1.0 score, no recovery); free activity (0 score, free activity; 0.5 score, reduced activity; 1.0 score, stimulating to be active; 2.0 score, stimulation is also inactive). mNSS scores were the sum of the above indexes.

### Beam Walking Test

All animals were trained for 1 week before the MCAO surgery. The balance beam used in this study had 2 cm width and 100 cm length. All animals can smoothly through the balance beam before surgery. After the surgery, the beam walking tests were evaluated as follows: 0 score, smoothly through the balance beam without tumble; 1.0 score, smoothly cross the balance beam and less than 50% of the way with slip feet; 2.0 score, smoothly cross the balance beam and more than 50% of the way with slip feet; 3.0 sore, cross the balance beam but the ipsilateral limb does not help move forward; 4.0 score, cannot cross the balance beam but can balance on it; 5.0 score, falling from the balance beam.

### Image Quantification and Statistical Analysis

The quantitation of positive cells and positive score by immunostaining were counted by Image J 1.5 software (Wayne Rasband, NIH, USA). The line graphs were prepared in SigmaPlot 10.0 software (Systat Software Inc., San Jose, California, USA); the histograms were prepared in GraphPad Prism 7.0 statistic software (GraphPad Software, Inc., La Jolla, CA, USA). All data were shown as mean ± SEM. Statistical analyses were performed in the SPSS 11.0 software (SPSS Inc., Chicago, IL, USA). Two-tailed Student’s *t* test was used in comparison between two groups. One-way ANOVA followed by post hoc Tukey-Kramer tests was used in comparison among groups. *P* < 0.05 was considered statistically significant.

## Results

### Generation of Cerebral Organoids

**Fig. 1:**
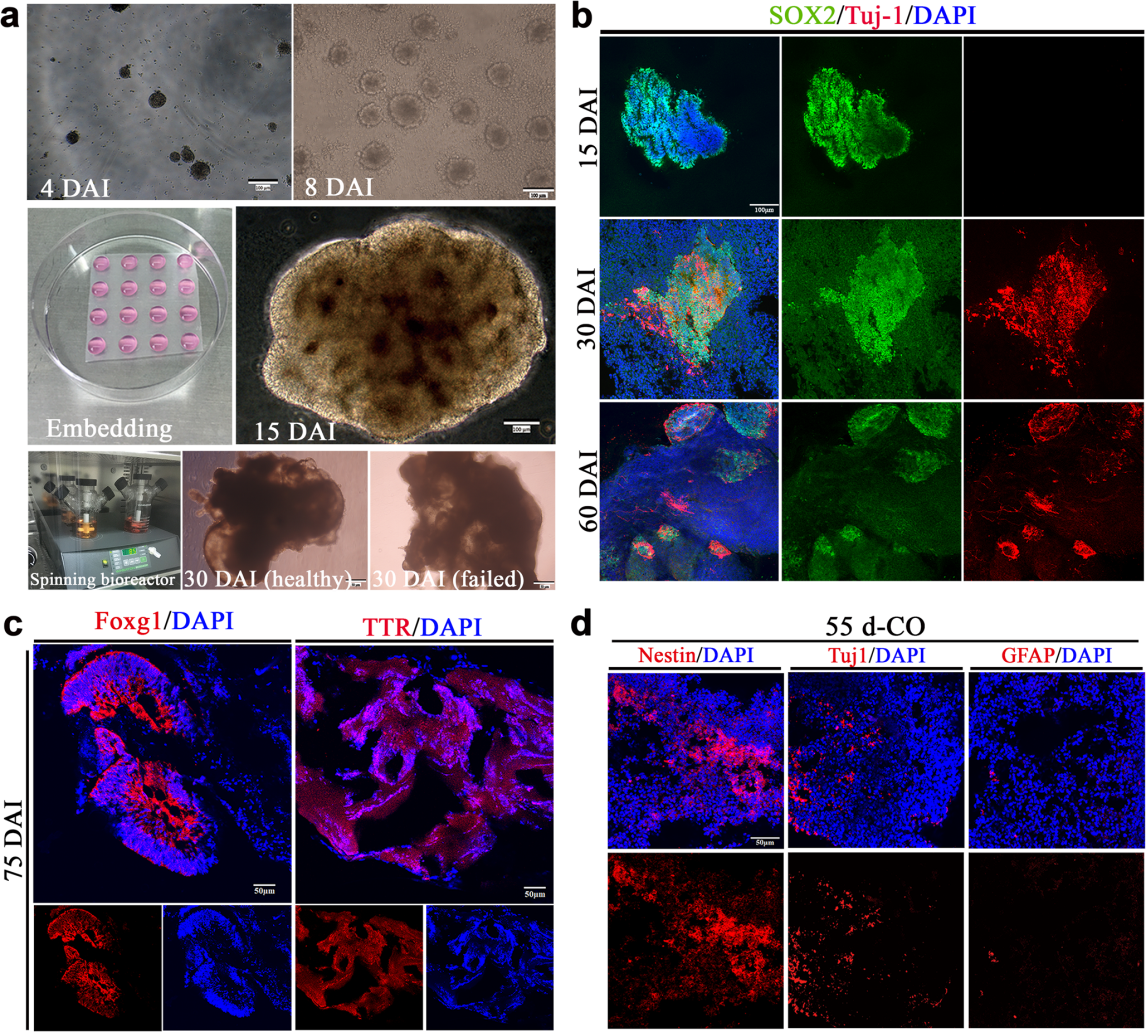
Generation of cerebral organoids (COs). **a** Schematic diagram of COs development. The initial formed embryonic bodies (EBs) in the low-attachment plate at 4 days after induction (DAI) from human embryonic stem cells H1. EBs at 8 DAI with evidence of ectodermal differentiation consisting of brightened surface and relative dark center in the tissue. The healthy EBs showed a smooth surface. After Matrigel embedding for stationary culture of expanding neuroepithelial buds, well-defined polarized neuroepithelium-like structures resembled neural tubes at 15 DAI. Then, cerebral tissues were transferred into the spinning bioreactor for further culture. Here are examples of healthy and failed COs at 30 DAI, respectively. **b** Immunostaining of SOX2 (green, neural progenitor cells marker) and Tuj1 (red, neurons marker) for cultured cerebral tissues at 15, 30, and 60 DAI. **c** Immunostaining of COs at 75 DAI with forebrain marker Foxg1 (red) and choroid plexus marker TTR (red). **d** Immunostaining of COs at 55 days with neural stem cells (Nestin), neurons (Tuj-1), and astrocytes (GFAP). DAPI labels nuclei (blue). All scale bars are as shown

COs were generated from human embryonic stem cells through germ layer differentiation, neural induction, formation of polarized neuroepithelium-like structures in the Matrigel droplets, and further growth with characteristics of fluid-filled cavity in the spinning bioreactor (Fig. 1a). With the prolongation of induction time, COs showed neural identity with positive expression of NPCs (SOX2) and gradually appeared neuronal identity with positive expression of neurons (Tuj-1) (Fig. 1b), indicating continuously neural differentiation during the in vitro culture. As expected, COs at 75 days had brain regional identities with expression of forebrain (Foxg1) and choroid plexus (TTR) (Fig. 1c), indicating the successful generation of COs. Here, we cultured COs at 55 days as transplantation donor for stroke study. COs at 55 days showed positive expression of NSCs, neurons and astrocytes, and NSCs expressed predominantly (Fig. 1d).

### COs Transplantation Reduces Brain Damage Volume and Improves Neurological Motor Function After Stroke

**Fig. 2:**
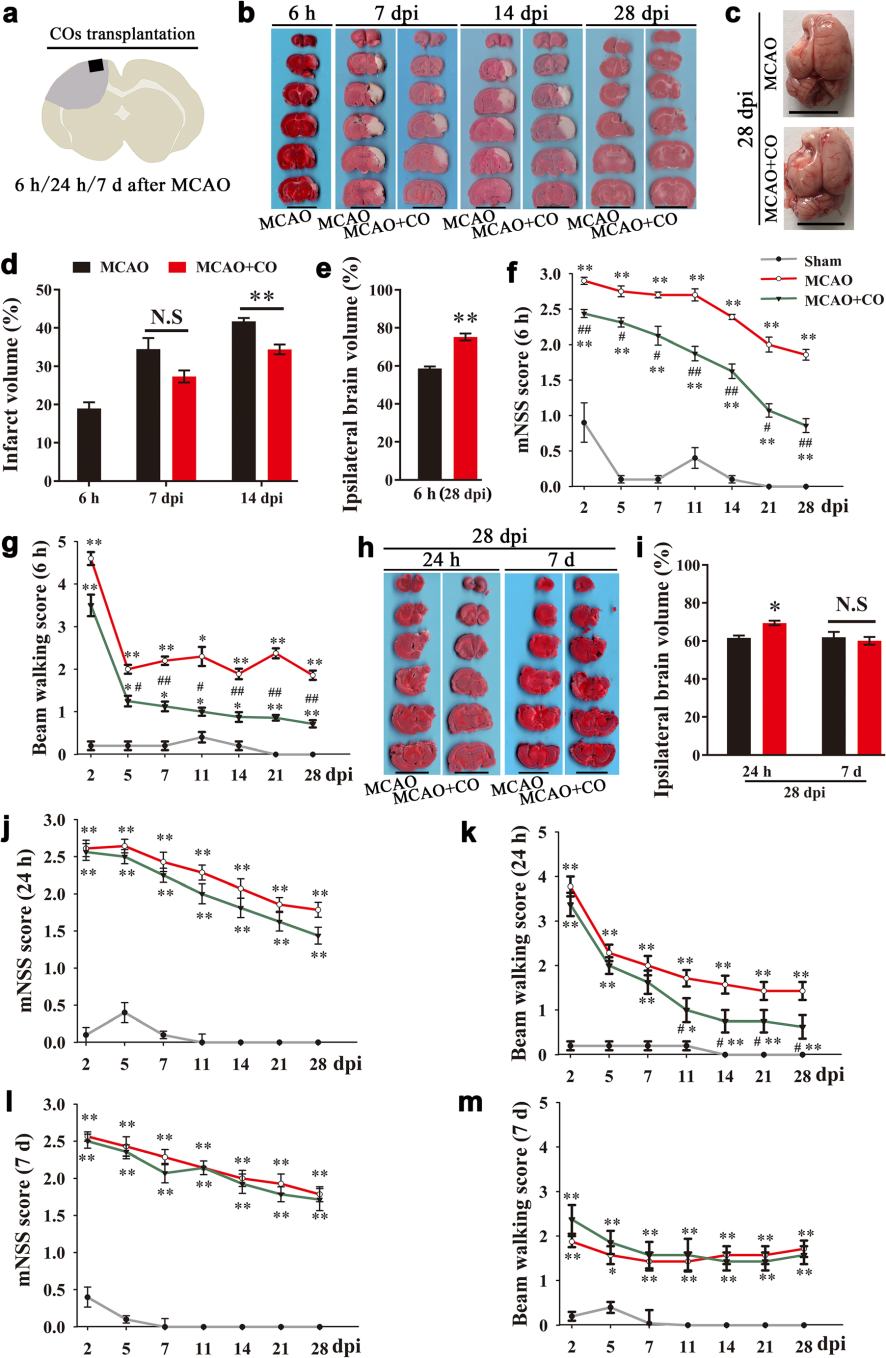
COs transplantation reduces brain damage volume and improves neurological motor function in the rat MCAO model. **a** Illustration for COs transplantation after MCAO. Two COs were transplanted into the cavity at 6 h, 24 h, or 7 days after MCAO. **b** TTC stained representative images of brain sections at 6 h after MCAO, and 7, 14, and 28-day post-implantation (dpi) in the model of COs transplantation at 6 h after MCAO. Scale bar: 1 cm. **c** Representative images of whole rat brain at 28 dpi in the model of COs transplantation at 6 h after MCAO. Scale bar: 1 cm. **d** Quantitative analysis of infarct volume in the model of COs transplantation at 6 h after MCAO. ^**^P < 0.01. N.S, no significant. *n* = 4 in each group. **e** Quantitative analysis of ipsilateral brain volume at 28 dpi in the model of COs transplantation at 6 h after MCAO. *n* = 8 in each group. ^**^P < 0.01. **f**, **g** Rat mNSS score and beam walking score were recorded at 2, 5, 7, 11, 14, 21, and 28 dpi in the model of COs transplantation at 6 h after MCAO. *n* = 8 in each group. ^*^P < 0.05, ^**^P < 0.01 versus Sham group; ^#^*P* < 0.05, ^##^*P* < 0.01 versus MCAO group. **h**, **i** Representative images and quantitation of brain infarction at 28 dpi in the model of COs transplantation at 24 h or 7 days after MCAO. Scale bar: 1 cm. ^*^P < 0.05. N.S, not significant. *n* = 8 in each group. **j**–**m** Rat mNSS score and beam walking score were recorded at 2, 5, 7, 11, 14, 21, and 28 dpi in the model of COs transplantation at 24 h or 7 days after MCAO. ^*^P < 0.05, ^**^P < 0.01 versus Sham group; ^#^*P* < 0.05 versus MCAO group. *n* = 8 in each group. All data are shown as mean ± SEM

Rat middle cerebral artery occlusion (MCAO) model of ischemic stroke was prepared for COs transplantation study (Fig. 2a). The final infarct volume was more than 40% at 14 days after MCAO (41.72 ± 0.88%, Fig. 2b, d). There was gradually increased infarct volume from 6 h to 14 days after MCAO (Fig. 2b, d). The infarct tissue in the ipsilateral brain eventually disappeared and formed a cavity at 28 days after MCAO (Fig. 2c). COs transplantation at 6 h after MCAO showed the decreased trend of infarct volume at 7-day post-implantation (dpi), and significantly reduced infarct volume at 14 dpi as compared to MCAO group (34.44 ± 1.30% vs. 41.72 ± 0.88%, Fig. 2d). COs transplantation preserved more survival brain parenchyma with smaller void in the ipsilateral cortex (Fig. 2c), with 75.22% ipsilateral brain volume as compared to 58.73% of MCAO group at 28 dpi (Fig. 2e). With examination of neurological motor function at 2, 5, 7, 11, 14, 21, and 28 dpi, COs transplantation at 6 h after MCAO improved neurological function and beam walking performance from 2 to 5 dpi, respectively, when compared to MCAO group (Fig. 2f, g).

Further, we would like to examine whether it is feasible to expand therapeutic time window for COs transplantation to 24 h, or even 7 days after MCAO. Of note, COs transplantation at 24 h after MCAO significantly rescued brain damage with more ipsilateral brain volume than MCAO group at 28 dpi (69.58 ± 1.20% vs. 61.68 ± 1.30%, Fig. 2h, i). There were also significant improvement of beam walking performance and better recovery trend of neurological function in the COs transplantation group at 24 h after MCAO as compared to MCAO group (Fig. 2j, k). Meanwhile, we examined brain damage volume and neurological motor function of COs transplantation at 7 days after MCAO. There was no observed benefit in the COs transplantation group at 7 days after MCAO (Fig. 2h, i, l, m).

### Cells from Transplanted COs Survive and Vascularize with the Host Brain After Stroke

As COs transplantation significantly decreased brain damage volume and improved neurological motor function after stroke, we further explored the mechanisms underlying COs transplantation at 6 h after MCAO. First of all, we examined cell survival of transplanted COs by immunostaining of human cytoplasmic marker STEM121 and HE staining in the COs transplantation group (Fig. S1a–c). Cells from transplanted COs survived in the transplantation periphery of ipsilateral cortex, and the cell number showed gradually decreased trend from 7 to 28 dpi (Fig. S1a, b). HE staining also showed cells from transplanted COs distributed throughout the transplantation cavity with gradually decreased trend from 7 to 28 dpi (Fig. S1c). Previous study reported growth of vascular network between grafted COs and host brain [11]; we also observed vascularization between transplanted COs and host brain by co-immunostaining of human cytoplasmic marker STEM121 and endothelial cell markers CD31 and CD34 at 7 and 28 dpi (Fig. S1d). The degree of vascularization at 28 dpi was relatively higher than that at 7 dpi (Fig. S1d). The successful vascularization plays a vital role for the long-time cell survival of transplanted COs in the host brain.

### Cells from Transplanted COs Show the Potential of Multilineage Differentiation to Mimic In Vivo Cortical Development, Support Motor Cortex Region-Specific Reconstruction, Form Neurotransmitter Related Neurons and Achieve Synaptic Connection with Host Brain Via In Situ Differentiation and Cell Replacement After Stroke

**Fig. 3:**
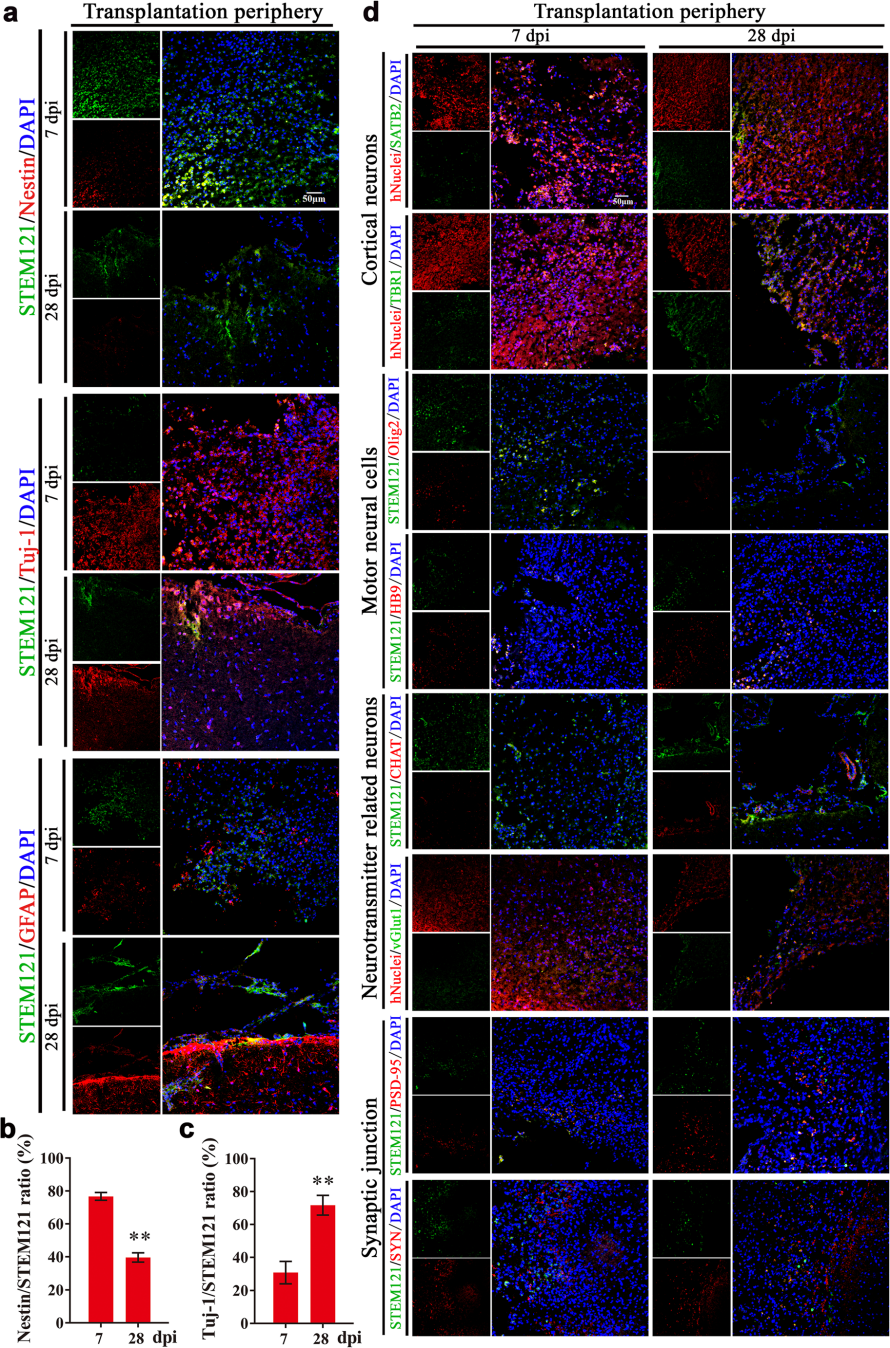
Cells from transplanted COs show the potential of multilineage differentiation to mimic in vivo cortical development, support motor cortex region-specific reconstruction, and achieve synaptic connection with host brain via in situ differentiation and cell replacement in the transplantation periphery of ipsilateral cortex of rat MCAO model. **a** Representative images of in vivo differentiated COs graft with cortical developmental characteristic by immunostaining of human cytoplasmic marker STEM121 (green) and neural stem cells Nestin (red), neurons Tuj-1 (red), or astrocytes GFAP (red) at 7- and 28-day post-implantation (dpi). DAPI labels nuclei (blue). **b**, **c** Quantification of the percentage of Nestin^+^/STEM121^+^ and Tuj1^+^/STEM121^+^ cells in the in vivo differentiated COs graft. Immuno-stained positive cells in each group were counted with at least five random microscope fields per section in three rats with ten sections per animal. ^**^P < 0.01. All data are shown as mean ± SEM. **d** Representative images of in vivo differentiated COs with identity of cortical neurons, motor cortex region-specific neural cell linages, neurotransmitter related neurons, and synaptic junction with host brain at 7 and 28 dpi in the transplantation periphery of ipsilateral cortex. Immunostaining of human cytoplasmic marker STEM121 (green) or human nuclear marker hNuclei (red) with surface-layer neurons marker SATB2 (green), pre-plate/deep-layer neurons marker TBR1 (green), motor progenitor cells marker Olig2 (red), motor neurons marker HB9 (red), cholinergic neurons marker Chat (red), glutamatergic neurons marker vGlut1 (green), postsynaptic marker PSD-95 (red), and presynaptic marker synaptophysin (SYN, red), showing in situ differentiation and cell replacement of transplanted COs in the transplantation periphery of ipsilateral cortex. DAPI labels nuclei (blue). All scale bars are as shown

Transplanted cells support brain region-specific reconstruction, functional replacement, and neural connection [27, 28]. COs at 55 days contain diverse neural cells with numerous NSCs (Fig. 1d), the potential of multilineage differentiation of COs after transplantation in stroke model is still unknown. Firstly, we examined differentiation trend of in vivo transplanted COs. With immunostaining of human cells derived NSCs (STEM121^+^/Nestin^+^), neurons (STEM121^+^/Tuj1^+^) and astrocytes (STEM121^+^/GFAP^+^), transplanted COs showed gradually increased expression of neurons and astrocytes and decreased expression of NSCs from 7 to 28 dpi (Fig. 3a–c), supporting in vivo differentiation and maturation of transplanted COs that resembled brain cortical development.

As the transplantation site was in the motor cortex, we further examined whether cells from transplanted COs differentiated with identity of cortical layer and even motor neural cell linage to support motor cortex region-specific reconstruction. Combined with human nuclear marker hNuclei, cells from transplanted COs showed positive expression of pre-plate/deep-layer neurons (TBR1) and late-born surface-layer neurons (SATB2) (Fig. 3d). The expression of late-born surface-layer neurons (SATB2) in the transplanted COs gradually increased from 7 to 28 dpi (Fig. 3d), suggesting some cells from transplanted COs gradually integrated into cortical layer over time. Meanwhile, we explored motor neural cell linages of transplanted COs by immunostaining of motor progenitor cells (Olig2) and motor neurons (HB9) with human cytoplastic marker STEM121. Transplanted COs showed decreased expression of STEM121^+^/Olig2^+^ cells and increased expression of STEM121^+^/HB9^+^ cells in the transplantation periphery of ipsilateral cortex, demonstrating some cells from transplanted COs differentiated from motor progenitor cells into motor neurons to support motor cortex-region specific reconstruction over time (Fig. 3d).

Further, we examined whether there were mature neurons in transplanted COs that regulated neurotransmitters release in the central neural system. We detected the positive expression of cholinergic marker Chat and glutamatergic marker vGlut1 in transplanted COs (Fig. 3d). Combined with extensive expression of neurons in transplanted COs (Fig. 3a), the positive expression of Chat and vGlut1 supported the formation of cholinergic and glutamatergic neurons in the transplanted COs, which are important for regulating neurotransmitters release. Meanwhile, we found that there were extensive expression of postsynaptic marker postsynaptic density protein 95 (PSD-95) and presynaptic marker synaptophysin (SYN) in the transplantation periphery, and some of them showed gradually increased overlap with cells from transplanted COs from 7 to 28 dpi (Fig. 3d), providing direct evidence for synaptic connection between transplanted COs and host brain.

### Cells from Transplanted COs Migrate into Host Brain After Stroke

**Fig. 4:**
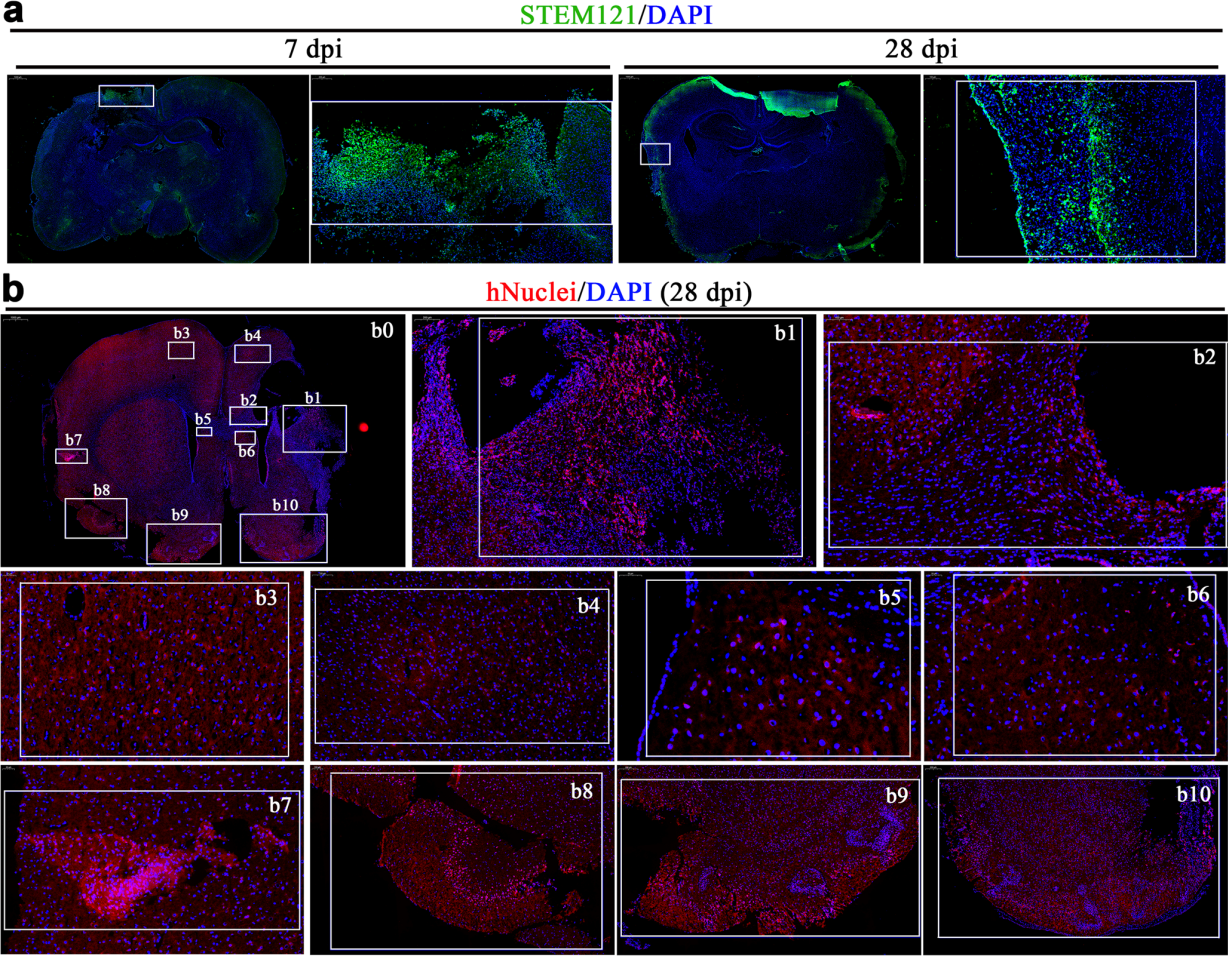
Cells from transplanted COs show extensive migration into host brain in the rat MCAO model. **a** Representative images of whole brain scan at 7- and 28-day post-implantation (dpi) in the COs transplantation group. STEM121 (green), human cytoplasmic marker. Tissues inside the rectangle frame indicate the transplanted COs in the host brain, wherein the right image is the high-magnification view of boxed area in the left image. **b** Representative images of whole brain scan at 28 dpi in the COs transplantation group. **b0** Images showed overall view of distribution of cells from transplanted COs at 28 dpi. **b1** Images showed cells from transplanted COs in the host brain with robust migration into cortical region. **b2** Images showed robust migration of cells from transplanted COs into host brain along corpus callosum. **b3–4** Images showed the migration of cells from transplanted COs into ipsilateral and contralateral cortex. **b5–6** Images showed the migration of cells from transplanted COs into ipsilateral and contralateral subventricular zone. **b7**–**10** Images showed the migration of cells from transplanted COs into ipsilateral and contralateral cortex with obvious neural stem cells pool. DAPI labels nuclei (blue). All scale bars are as shown

In addition to in situ differentiation and cell replacement of transplanted COs, we found cells from transplanted COs show extensive distribution in the host brain at 7 and 28 dpi, including but not limited to transplantation cavity (Fig. 4a). Cells from transplanted COs migrated into different brain regions of host brain, including but not limited to ipsilateral and contralateral cortex (Fig. 4b1, b3-4, b7–10) and SVZ (Fig. 4b2, b5–6). Cells from transplanted COs migrated from transplantation periphery into deep brain regions along corpus callosum at 28 dpi (Fig. 4b1–2). Notably, considerable cells from transplanted COs densely lied in some areas of host brain at 28 dpi (Fig. 4b7–10), suggesting that cells from transplanted COs may form neural cell pool in the host brain. In combination with STEM121^+^/PSD-95^+^ and STEM121^+^/SYN^+^ expression in transplanted COs (Fig. 3d), the extensive distribution of human cells in the host brain revealed extensive synaptic connection between transplanted COs and host brain.

### COs Transplantation Enhances Neurogenesis with Predominantly Exogenous Neurogenesis in the Transplantation Periphery of Ipsilateral Cortex and Predominantly Endogenous Neurogenesis in the Hippocampus and Subventricular Zone After Stroke

It is generally agreed that the restorative process of stroke is associated with multiple mechanisms including, but not limited to, neurogenesis, neural plasticity, and angiogenesis [2, 29]. Neurogenesis is an endogenous process involving proliferation, differentiation, and migration of NPCs/NSCs in the hippocampal subgranular zone (SGZ) and subventricular zone (SVZ), which is important for replacing gradual neuron loss and maintaining specific neuronal function throughout life [30, 31].

**Fig. 5:**
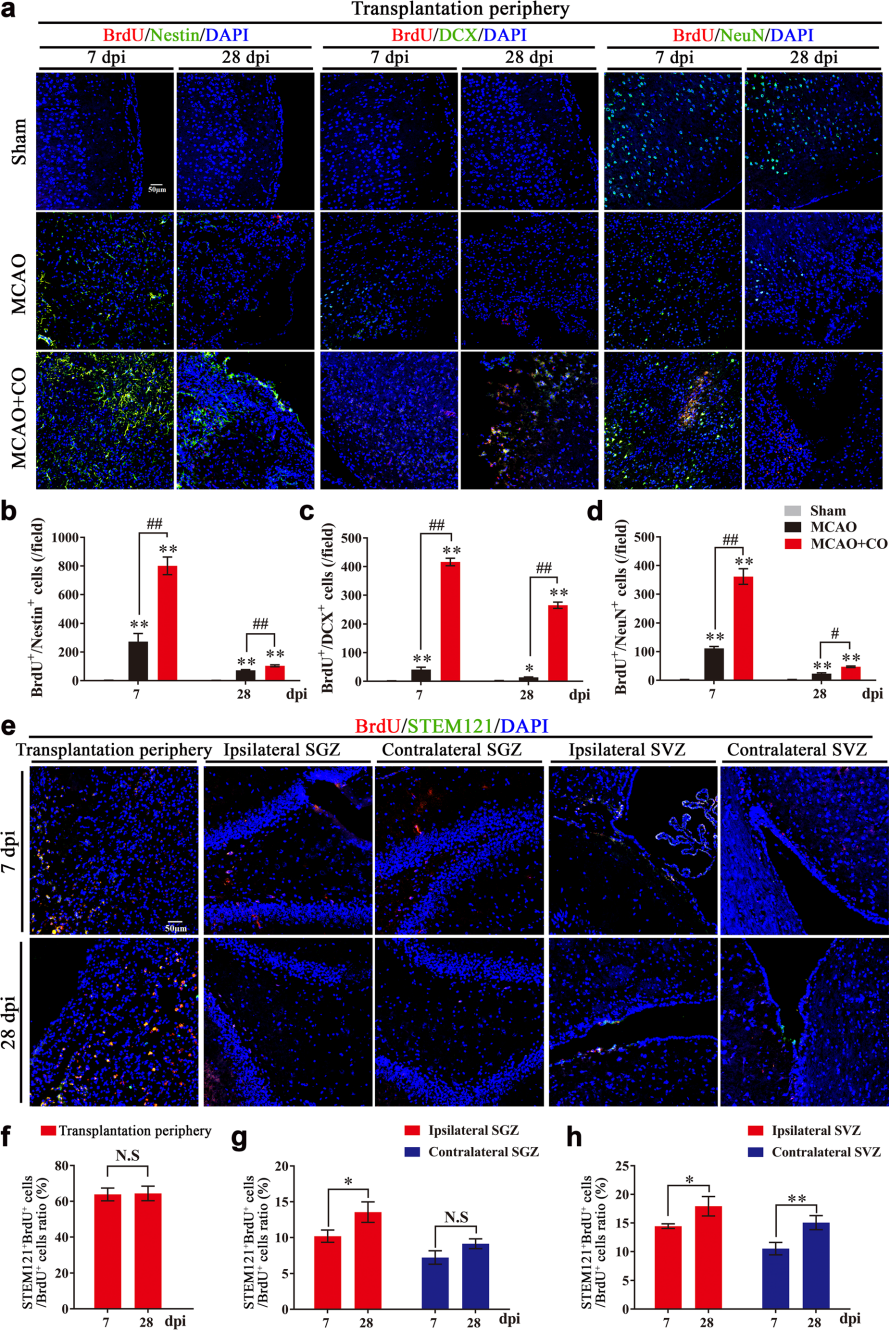
COs transplantation enhances neurogenesis with predominantly exogenous neurogenesis in the transplantation periphery of ipsilateral cortex and predominantly endogenous neurogenesis in the hippocampal subgranular zone (SGZ) and subventricular zone (SVZ) of rat MCAO model. **a** Representative images of neurogenesis in the rat transplantation periphery of ipsilateral cortex by immunostaining of proliferated neural stem cells (BrdU^+^/Nestin^+^), migrated newborn neurons (BrdU^+^/DCX^+^), and finally differentiated mature neurons (BrdU^+^/NeuN^+^) at 7- and 28-day post-implantation (dpi) in Sham, MCAO, and COs transplantation groups. DAPI labels nuclei (blue). **b**–**d** Quantitative analysis of neurogenesis by counting BrdU^+^/Nestin^+^, BrdU^+^/DCX^+^ and BrdU^+^/NeuN^+^ cells per field at 7 and 28 dpi in the rat transplantation periphery of ipsilateral cortex. ^*^P < 0.05, ^**^P < 0.01 versus Sham group; ^#^*P* < 0.05, ^##^*P* < 0.01. **e** Representative images of exogenous neurogenesis in the rat transplantation periphery of ipsilateral cortex, ipsilateral, and contralateral SGZ and SVZ by immunostaining of human-derived proliferation cells (BrdU^+^/STEM121^+^) at 7 and 28 dpi in the COs transplantation group. DAPI labels nuclei (blue). **f**–**h** Quantitative analysis of exogenous neurogenesis by counting BrdU^+^/STEM121^+^ cells per field at 7 and 28 dpi in the rat transplantation periphery of ipsilateral cortex, ipsilateral and contralateral SGZ and SVZ of COs transplantation group. ^*^P < 0.05, ^**^P < 0.01. N.S, not significant. Immuno-stained positive cells in each group were counted with at least five random microscope fields per section in three rats with ten sections per animal. All scale bars are as shown. All data are shown as mean ± SEM

Firstly, we examined neurogenesis mediated by COs transplantation after stroke. Compared to MCAO group, COs transplantation significantly increased the expression of BrdU^+^/Nestin^+^ proliferated NSCs, BrdU^+^/DCX^+^ migrated newborn neurons and BrdU^+^/NeuN^+^ differentiated mature neurons at 7 and 28 dpi in the rat transplantation periphery of ipsilateral cortex (Fig. 5a–d). Similarly, the number of BrdU^+^/Nestin^+^ and BrdU^+^/DCX^+^ cells at 7 and 28 dpi in the ipsilateral and contralateral hippocampal SGZ and SVZ of COs transplantation group were also more than these in the MCAO group (Figs. S2 and S3).

The enhanced neurogenesis may result from endogenous and exogenous neurogenesis, we wondered the role of COs transplantation on endogenous and exogenous neurogenesis in the host brain. With immunostaining of BrdU^+^/STEM121^+^ human-derived proliferation cells, the proportion of BrdU^+^/STEM121^+^ cells in the total BrdU^+^ cells were more than 60% in the transplantation periphery of ipsilateral cortex (Fig. 5e, f), less than 15% in the ipsilateral and contralateral SGZ (Fig. 5e, g) and less than 20% in the ipsilateral and contralateral SVZ (Fig. 5e, h) at 7 and 28 dpi in the transplantation group. Therefore, COs transplantation promoted predominantly exogenous neurogenesis in the transplantation periphery of ipsilateral cortex and predominantly endogenous neurogenesis in the SGZ and SVZ after stroke.

### COs Transplantation Promotes Synaptic Reconstruction, Axonal Regeneration, and Angiogenesis After Stroke

**Fig. 6:**
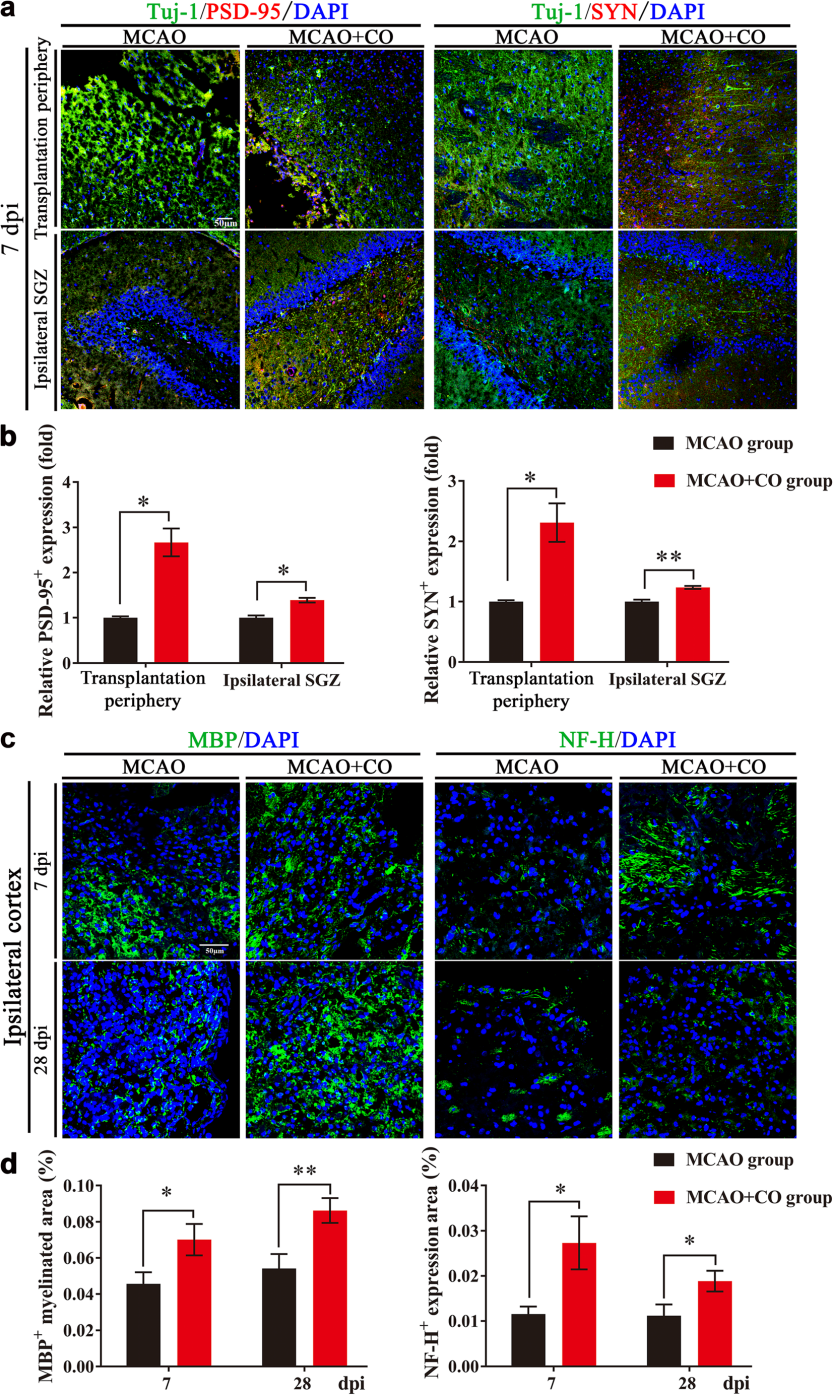
COs transplantation enhances synaptic reconstruction and axonal regeneration in the rat MCAO model. **a** Representative images of synaptic reconstruction at 7- and 28-day post-implantation (dpi) by immunostaining of synaptic connection-related proteins (Tuj-1^+^/PSD-95^+^, Tuj-1^+^/SYN^+^) in the rat transplantation periphery of ipsilateral cortex and ipsilateral hippocampal SGZ of MCAO and COs transplantation groups. Tuj-1, neuronal marker; PSD-95, postsynaptic marker; synaptophysin (SYN), presynaptic marker. DAPI labels nuclei (blue). **b** Quantitative analysis of relative PSD-95 and SYN expression at 7 and 28 dpi in the rat transplantation periphery of ipsilateral cortex and ipsilateral hippocampal SGZ. ^*^P < 0.05, ^**^P < 0.01 versus MCAO group. **c** Representative images of remyelination and axonal regeneration by immunostaining of myelin basic protein (MBP, green) and Neurofilament-H (NF-H, green) at 7 and 28 dpi in the ipsilateral cortex of MCAO and COs transplantation groups. DAPI labels nuclei (blue). **d** Quantitative area ratio of MBP^+^ myelinated area and NF-H^+^ expression per field at 7 and 28 dpi in the ipsilateral cortex. ^*^P < 0.05, ^**^P < 0.01. All scale bars are as shown. All data are shown as mean ± SEM

Postsynaptic marker PSD-95 and presynaptic marker SYN are synaptic connection-related proteins that are important for neural plasticity. With immunostaining of Tuj-1^+^/PSD-95^+^ and Tuj-1^+^/SYN^+^ expression, we found that COs transplantation increased the expression of PSD-95 and SYN at 7 dpi in the transplantation periphery of ipsilateral cortex and ipsilateral SGZ of COs transplantation group, compared with MCAO group (Fig. 6a, b), providing evidence for enhanced synaptic reconstruction by COs transplantation after stroke. Further, we examined myelin and axonal integrity by immunostaining of myelin basic protein (MBP) and Neurofilament-H (NF-H) after COs transplantation. COs transplantation upregulated MBP^+^ myelinated area and promoted NF-H^+^ axonal regeneration as compared to MCAO group (Fig. 6c, d). As oligodendrocyte survival is closely related to the production of myelin sheaths and preservation of axon function [32], we introduced adult oligodendrocyte-precursor cell (OPCs) marker platelet-derived growth factor receptor α (PDGFRα) and oligodendrocyte-lineage cell marker Nkx2.2 to explore the role of COs transplantation on oligodendrogenesis after stroke. Compared to MCAO group, COs transplantation had higher expression of BrdU^+^/PDGFRα^+^ proliferated OPCs and BrdU^+^/ Nkx2.2^+^ proliferated oligodendrocyte-lineage cells in the ipsilateral cortex (Fig. S4). Therefore, COs transplantation not only enhanced synaptic reconstruction, but also promoted remyelination, axonal regeneration, and oligodendrogenesis after stroke.

**Fig. 7:**
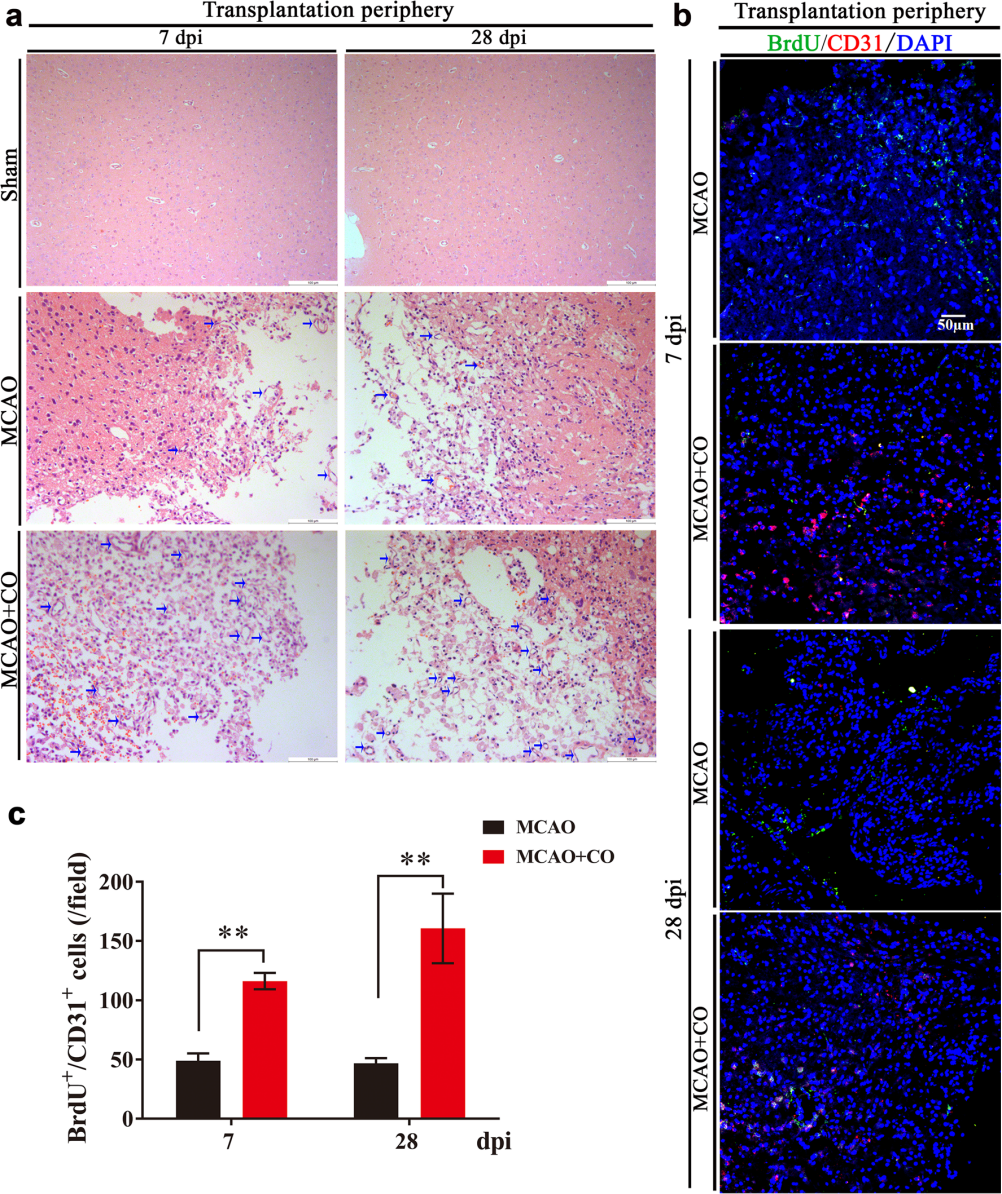
COs transplantation promotes angiogenesis in the transplantation periphery of ipsilateral cortex of rat MCAO model. **a** Representative images of vessel density by HE staining at 7- and 28-day post-implantation (dpi) in the rat transplantation periphery of ipsilateral cortex of Sham, MCAO, and COs transplantation groups. The vessels were shown as indicated arrows. All scale bars are as shown. **b** Representative images of angiogenesis at 7 and 28 dpi by immunostaining of new formed blood vessels (BrdU^+^/CD31^+^) in the rat transplantation periphery of ipsilateral cortex of MCAO and COs transplantation groups. BrdU, proliferation marker; CD31, endothelial marker. DAPI labels nuclei (blue). All scale bars are as shown. **c** Quantitative analysis of BrdU^+^/CD31^+^ new formed blood vessels at 7 and 28 dpi in the rat transplantation periphery of ipsilateral cortex of MCAO and COs transplantation groups. Immuno-stained positive cells in each group were counted with at least five random microscope fields per section in three rats with ten sections per animal. ^**^P < 0.01 versus MCAO group. All data are shown as mean ± SEM

At the same time, with the observation of HE-stained brain slice, we found that COs transplantation group showed higher vessel density in the transplantation periphery of ipsilateral cortex at 7 and 28 dpi, as compared to MCAO group (indicated by arrows, Fig. 7a), we wondered whether COs transplantation promoted angiogenesis after stroke. With immunostaining of BrdU^+^/CD31^+^ new formed vessels, the expression of BrdU^+^/CD31^+^ cells in the COs transplantation group was more than that in MCAO group at 7 and 28 dpi (116.14 ± 6.92 vs. 49.00 ± 6.27 at 7 dpi, 160.71 ± 29.39 vs. 46.86 ± 4.44 at 28 dpi, Fig. 7b, c), indicating that COs transplantation did promote angiogenesis after stroke.

### COs Transplantation Decreases Neural Apoptosis and Rescues More Survival Neurons After Stroke

Due to neural apoptosis and neuroinflammation existing in the whole brain after stroke, we explored the effect of COs transplantation on neural apoptosis and neuroinflammation after MCAO. With TUNEL staining for cell apoptosis and Nissl’s staining for survival neurons, we found that COs transplantation significantly decreased TUNEL positive cells and increased Nissl’s positive neurons at 7 and 28 dpi in the transplantation periphery of ipsilateral cortex after MCAO (Fig. S5), demonstrating decreased neural apoptosis and more survival neurons mediated by COs transplantation. With immunostaining of pro-inflammatory cytokines TNF-α (tumor necrosis factor α) and IL-1 β (interleukin-1 β), phagocytosis marker CD68 and infiltrated neutrophils MPO-1 in the transplantation periphery of Sham, MCAO, COs transplantation groups at 7 and 28 dpi, the level of neuroinflammation in the MCAO and COs transplantation groups significantly was higher than Sham group (Fig. S6). Compared to MCAO group, there was no difference of neuroinflammation in the COs transplantation group (Fig. S6). Although COs transplantation had no benefit on neuroinflammation, no aggravated neuroinflammation was observed after COs transplantation, demonstrating the good compatibility of transplanted COs in the host brain without observed immune rejection after stroke.

Brain astrocytes are beneficial for sealing the site of injury, remodeling the tissue, and controlling the local immune response. However, activated astrocytes also lead to excessive astrogliosis and formation of a wall-like structure described as glial scar, which are detrimental for neurite outgrowth and neurogenesis [12, 13]. We further examined whether transplanted COs took part in glia scar formation due to positive expression of astrocytes in transplanted COs (Figs. 3a and S7a). In the infarct border zone, cells from transplanted COs had positive expression of Neurocan, a chondroitin sulfate proteoglycan that is related to glia scar formation (Fig. S7a). Moreover, transplanted COs upregulated Neurocan^+^ expression as compared to MCAO group, though there was no difference on GFAP expression between MCAO and COs transplantation groups (Fig. S7b, c), suggesting the participation of transplanted COs in glia scar formation.

## Discussion

The study gives the first demonstration for efficacy and mechanism of COs transplantation in stroke. The major findings of the study are as follows: transplantation of COs at 6 h or 24 h after MCAO significantly reduces brain damage volume and improves neurological motor function; cells from transplanted COs have the potential of multilineage differentiation and migrate into different brain regions along corpus callosum; COs transplantation enhances neurogenesis, synaptic reconstruction, axonal regeneration, and angiogenesis and decreases neural apoptosis with more survival neurons after stroke.

### The Therapeutic Time Window of COs Transplantation in Stroke

Therapeutic time window is an important parameter for preclinical transplantation studies [4, 33]. The therapeutic time window for only drug t-PA is within 4.5 h, and EVT is from 6 h of current guideline to potential 24 h with more careful selection criteria in ischemic stroke. So, we wondered the possible therapeutic time window for COs transplantation in stroke, as an important parameter for practical application. Notably, COs transplantation at 6 h or even 24 h after MCAO shows significant benefits in brain damage repair and functional motor function recovery, suggesting the expandable therapeutic time window of COs transplantation for stroke. Although there is no observed benefit for COs transplantation at 7 days after MCAO, it does not mean that it is not feasible for COs transplantation at 7 days after MCAO due to the transplantation site. Glial scar may have formed surrounding the transplantation site at 7 days after MCAO, which hinder the survival and migration of transplanted COs. If the transplantation site is changed, COs transplantation at 7 days after stroke may be effective. Meanwhile, the efficacy of COs transplantation within 7 days after stroke is still a question that needs to be further examined in the future study. Besides, we observed neurogenesis in the hippocampal CA1, CA2, and CA3 subregions of COs transplantation group (data not shown). Increasing studies have demonstrated specific roles of hippocampal subregions in distinct aspects of declarative memory, such as dentate gyrus (DG) for pattern separation [34], CA1/subiculum for temporal association memory [35], CA2 for social memory [36], and CA3 for pattern completion and one-trial contextual learning [37]. Therefore, the neurogenesis in the hippocampal CA1, CA2, and CA3 subregions mediated by COs transplantation may contribute to the improvement of learning and memory function after stroke and need to be investigated in the future study.

### The Mechanism of COs Transplantation in Stroke

The survival, differentiation, replacement and integration of transplanted cells in the host brain are the specific mechanisms for stem cell therapy [4]. The general mechanisms for stroke recovery are associated with neurogenesis, neural plasticity, and angiogenesis [2, 38]. Thus, the present study explored the above fields to illustrate underlying beneficial mechanisms of COs transplantation at 6 h after MCAO for the first time.

As with reported cell survival and vascularization of transplanted COs in the mouse brain [11, 12], we observed the similar results in our study. Given the vessels were derived from host brain [11], the source of vessels is not explored in our study. Considering transplanted cells have the potential of replacing damaged cells in the host brain [33, 39], we explored the potential of multilineage differentiation of transplanted COs in the transplantation periphery of stroke. Transplanted COs show increased expression of neurons and astrocytes and decreased expression of NSCs, resembling in vivo differentiation and maturation of brain cortical development. Cells from transplanted COs differentiate with identity of cortical layer neurons, motor progenitor cells, and motor neurons in the transplantation periphery of ipsilateral cortex. In combination with the transplantation site in the motor cortex, the formation of cortical layer neurons and motor neural cells in the transplanted COs supports motor cortex region-specific reconstruction, contributing to brain damage repair and neurological motor recovery. Besides, we found the formation of cholinergic and glutamatergic neurons in the transplanted COs that are important for the regulation of neurotransmitter release. Transplanted cells can form functional synaptic inputs into host brain [40, 41]. Along with extensive expression of synaptic connection-related proteins in the host brain, we found that some of them have gradually increased overlap with cells from transplanted COs, indicating synaptic connection between transplanted COs and host brain after stroke.

Considering the cell number of transplanted COs in the transplantation periphery of ipsilateral cortex decreases over time, we wondered whether it was due to gradual migration into host brain. As expected, cells from transplanted COs migrate into different brain regions along corpus callosum, including but not limited to ipsilateral and contralateral cortex and SVZ. Notably, cells from transplanted COs form neural cell pool in some areas of host brain, providing possibility for replacing gradually neuronal loss and repairing brain damage as a cell banking. In combination with extensive expression of synaptic connection-related proteins in the transplanted COs, the extensive distribution and integration of human cells in the host brain reveal extensive synaptic connection and axon projection from transplanted COs into host brain.

Transplanted cells have been reported to reduce cell death and provide growth/trophic support to enhance endogenous regeneration via paracrine action in the host brain [4, 41]. With examination of neurogenesis in the host brain, COs transplantation significantly enhances neurogenesis in the ipsilateral cortex, SGZ, and SVZ after MCAO. Due to the differentiation and migration of transplanted COs in the host brain, the enhanced neurogenesis may result from endogenous and exogenous neurogenesis. With observation of the role of COs transplantation on different brain regions after stroke, COs transplantation promotes predominantly exogenous neurogenesis in the transplantation periphery of ipsilateral cortex and predominantly endogenous neurogenesis in the SGZ and SVZ after MCAO. And, COs transplantation not only enhances synaptic reconstruction, remyelination, and axonal regeneration, but also promotes oligodendrogenesis after stroke, supporting reported evidence that axonal function is closely related to the preservation of myelin integrity and oligodendrocyte survival following ischemic white matter injury [32]. In the reported study of COs transplantation in the mice [11], neurons from transplanted COs have synchronized neural activity, which is similar to neuronal activity in postnatal mouse cortex [42], suggesting transplanted COs, at least in part, resemble the function of postnatal mouse neocortex. At the later stage of transplantation, there are synchronized firing neurons and local field potential in transplanted COs [11], providing evidence for generation of functional neuronal networks and functional connectivity between transplanted COs and host brain. Besides, COs transplantation promotes angiogenesis after stroke. The enhanced angiogenesis has the mutual benefit, where transplanted COs provide trophic support for vascular formation and new formed vessels support the survival of transplanted COs. And, the enhanced neurogenesis, angiogenesis, and synaptic reconstruction have positive correlation with improved neurological motor function by COs transplantation after stroke (data not shown), providing evidence for the benefits of neurogenesis, angiogenesis, and synaptic reconstruction on functional improvement after stroke.

Neural apoptosis and neuroinflammation have impact on the restorative process of stroke and play an important role in stem cell transplantation therapy [4, 43]. With TUNEL and Nissl’s staining in the transplantation periphery of ipsilateral cortex, COs transplantation significantly decreases neural apoptosis and rescues more survival neurons, which may benefit from synaptic reconstruction and angiogenesis mediated by COs transplantation. The migration of cells from transplanted COs into host brain also contributes to more survival neurons in the transplantation periphery. Although COs transplantation takes part in glia scar formation, there is no deleterious effect of neuroinflammation meditated by COs transplantation, showing the good compatibility of transplanted COs in the host brain. Recently, a study revealed that astrocyte scar formation aids rather prevents axon regeneration. Whether the glia scar formation contributes to axonal regeneration in our study of COs transplantation after stroke need to be explored in the future study.

In conclusion, with identification of therapeutic time window for COs transplantation in stroke, the study demonstrates the feasibility, efficacy, and underlying mechanisms of COs transplantation in stroke. This preliminary but promising study provides first-hand preclinical evidence for COs transplantation as a potential and effective intervention for stroke treatment.

## Electronic supplementary material

ESM 1 (DOCX 12.4 MB)
