# Supplementary material for: Cerebral Organoids Repair Ischemic Stroke Brain Injury
Source: Transl Stroke Res. 2019 Dec 30;11(5):983–1000. doi: 10.1007/s12975-019-00773-0 (PMC7496035; doi:10.1007/s12975-019-00773-0)
Supplement: Supplementary file 1 — (DOCX 12.4 MB) [file 12975_2019_773_MOESM1_ESM.docx]

**Translational Stroke Research**

**Supplementary Material**

**Cerebral organoids repair ischemic stroke brain injury**

Shu-Na Wang^1,#^, Zhi Wang^1,#^, Tian-Ying Xu^1^, Ming-He Cheng^1^, Wen-Lin Li^2^, Chao-Yu Miao^1,^*

^1^Department of Pharmacology, Second Military Medical University, 325 Guo He Road, Shanghai 200433, China

^2^Department of Cell Biology, Second Military Medical University, 800 Xiang Yin Road, Shanghai 200433, China

#These authors contributed equally to this work.

***Corresponding Author**

Chao-Yu Miao

MD, PhD, Professor and Chair

Department of Pharmacology

Second Military Medical University

325 Guo He Road, Shanghai 200433, China

Tel: +86 21 81871271, Fax: +86 21 65493951

Email: [cymiao@smmu.edu.cn](mailto:cymiao@smmu.edu.cn)

ORCID: 0000-0002-8176-3434

**The** **supplementary material includes:**

Supplementary Figure 1. Cells from transplanted COs survive and vascularize with the host brain in the rat MCAO model.

Supplementary Figure 2. COs transplantation enhances neurogenesis in the ipsilateral and contralateral hippocampal subgranular zone (SGZ) of rat MCAO model.

Supplementary Figure 3. COs transplantation enhances neurogenesis in the ipsilateral and contralateral subventricular zone (SVZ) of rat MCAO model.

Supplementary Figure 4. COs transplantation promotes oligodendrogenesis in the ipsilateral cortex of rat MCAO model.

Supplementary Figure 5. COs transplantation reduces neural apoptosis and increases neuronal survival in the infarct periphery of rat MCAO model.

Supplementary Figure 6. COs transplantation has no impact on neuroinflammation in the infarct periphery of rat MCAO model.

Supplementary Figure 7. Transplanted COs take part in the formation of glia scar in the infarct border zone of rat MCAO model.

Supplementary Table 1. Antibodies used in this study.

**
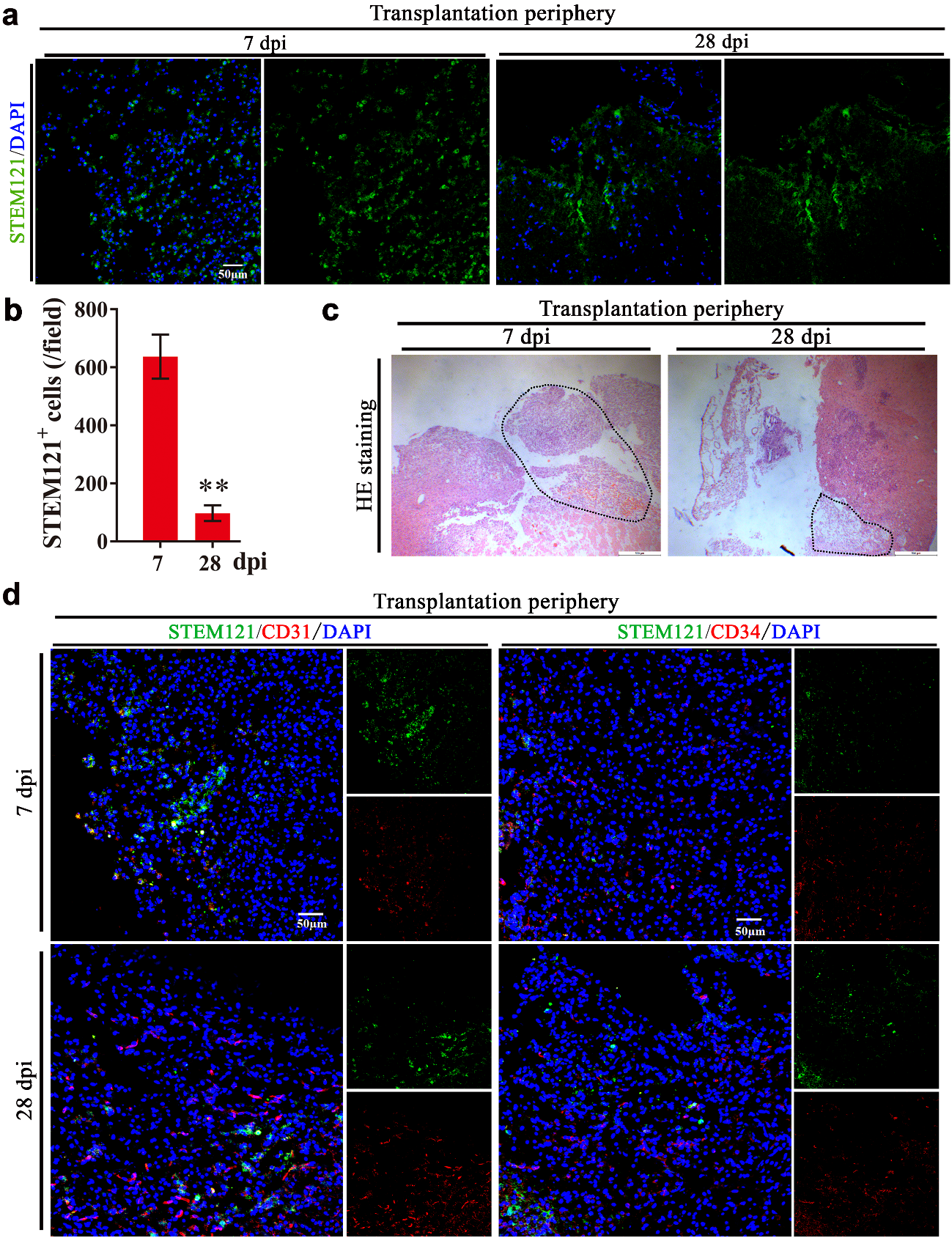
**

**Supplementary Figure 1. Cells from transplanted COs survive and vascularize with the host brain in the rat MCAO model.** (**a**) Representative images of COs survival at 7- and 28-day post-implantation (dpi) in the transplantation periphery of ipsilateral cortex by immunostaining of human cytoplasmic marker (STEM121, green) in the COs transplantation group. STEM121^+^ cells distributed throughout the transplantation cavity. DAPI labels nuclei (blue). (**b**) Quantitative analysis of STEM121^+^ cells per field in the COs transplantation group**.** Immuno-stained positive cells in each group were counted with at least five random microscope fields per section in three rats with ten sections per animal. All data are shown as mean ± SEM. ^**^P < 0.01. (**c**) Representative images of COs survival by HE staining in the rat ipsilateral cortex. The cavity was filled with transplanted COs (dotted black lines). n=3 animals in each group. (**d**) Representative images of vascularization between transplanted COs and host brain by immunostaining of human cytoplasmic marker STEM121 (green) with endothelial cells markers CD31 and CD34 (red) at 7 and 28 dpi. DAPI labels nuclei (blue). All scale bars are as shown.


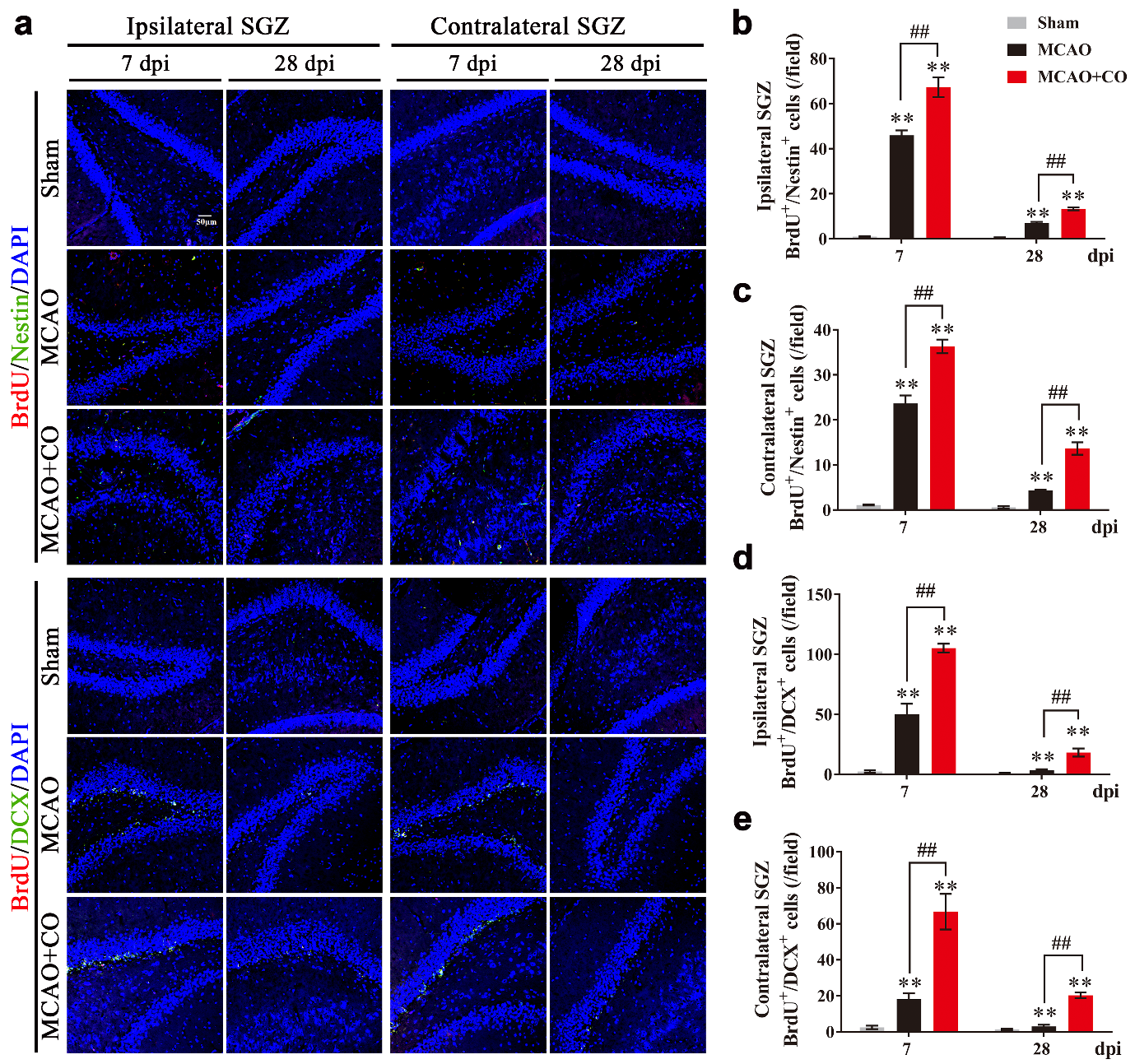


**Supplementary Figure 2. COs transplantation enhances neurogenesis in the ipsilateral and contralateral hippocampal subgranular zone (SGZ) of rat MCAO model.** (**a**) Representative images of neurogenesis at 7- and 28-day post-implantation (dpi) in the rat ipsilateral and contralateral hippocampal SGZ by immunostaining of proliferated neural stem cells (BrdU^+^/Nestin^+^, red and green respectively) and migrated newborn neurons (BrdU^+^/DCX^+^, red and green respectively) in Sham, MCAO and COs transplantation groups. DAPI labels nuclei (blue). All scale bars are as shown. (**b-e**) Quantitative analysis of neurogenesis by counting BrdU^+^/Nestin^+^ and BrdU^+^/DCX^+^ cells per field at 7 and 28 dpi in the rat ipsilateral and contralateral hippocampal SGZ. All groups had significant difference when compared to Sham group. Immuno-stained positive cells in each group were counted with at least five random microscope fields per section in three rats with ten sections per animal. ^**^*P* < 0.01 versus Sham group; ^##^*P* < 0.01 versus MCAO group. All data are shown as mean ± SEM.

**
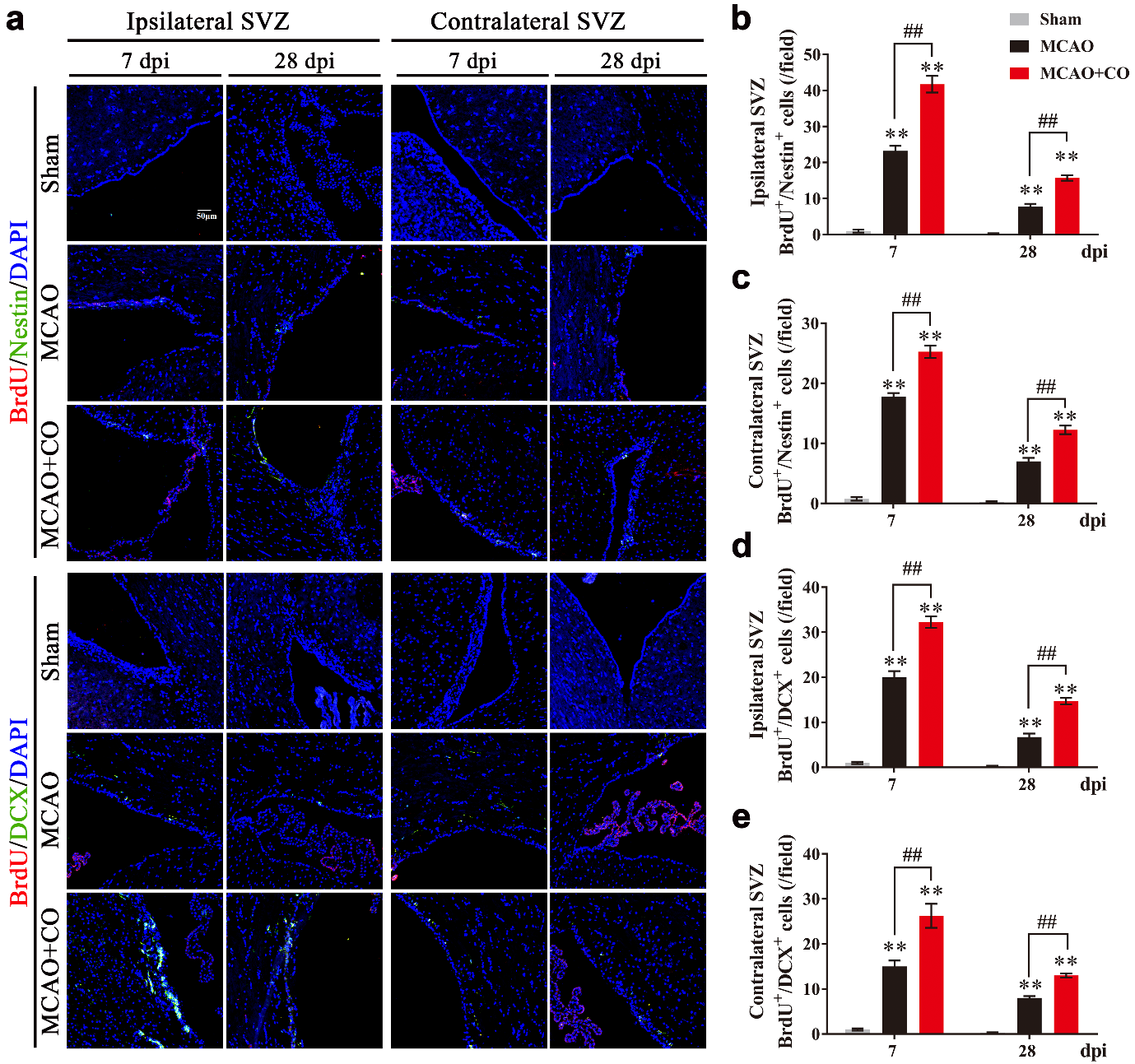
**

**Supplementary Figure 3. COs transplantation enhances neurogenesis in the ipsilateral and contralateral subventricular zone (SVZ) of rat MCAO model.** (**a**) Representative images of neurogenesis at 7- and 28-day post-implantation (dpi) in the rat ipsilateral and contralateral hippocampal SVZ by immunostaining of proliferated neural stem cells (BrdU^+^/Nestin^+^, red and green respectively) and migrated newborn neurons (BrdU^+^/DCX^+^, red and green respectively) in Sham, MCAO and COs transplantation groups. DAPI labels nuclei (blue). All scale bars are as shown. (**b-e**) Quantitative analysis of neurogenesis by counting BrdU^+^/Nestin^+^ and BrdU^+^/DCX^+^ cells per field at 7 and 28 dpi in the rat ipsilateral and contralateral hippocampal SVZ. All groups had significant difference when compared to Sham group. Immuno-stained positive cells in each group were counted with at least five random microscope fields per section in three rats with ten sections per animal. ^**^*P* < 0.01 versus Sham group; ^##^*P* < 0.01 versus MCAO group. All data are shown as mean ± SEM.


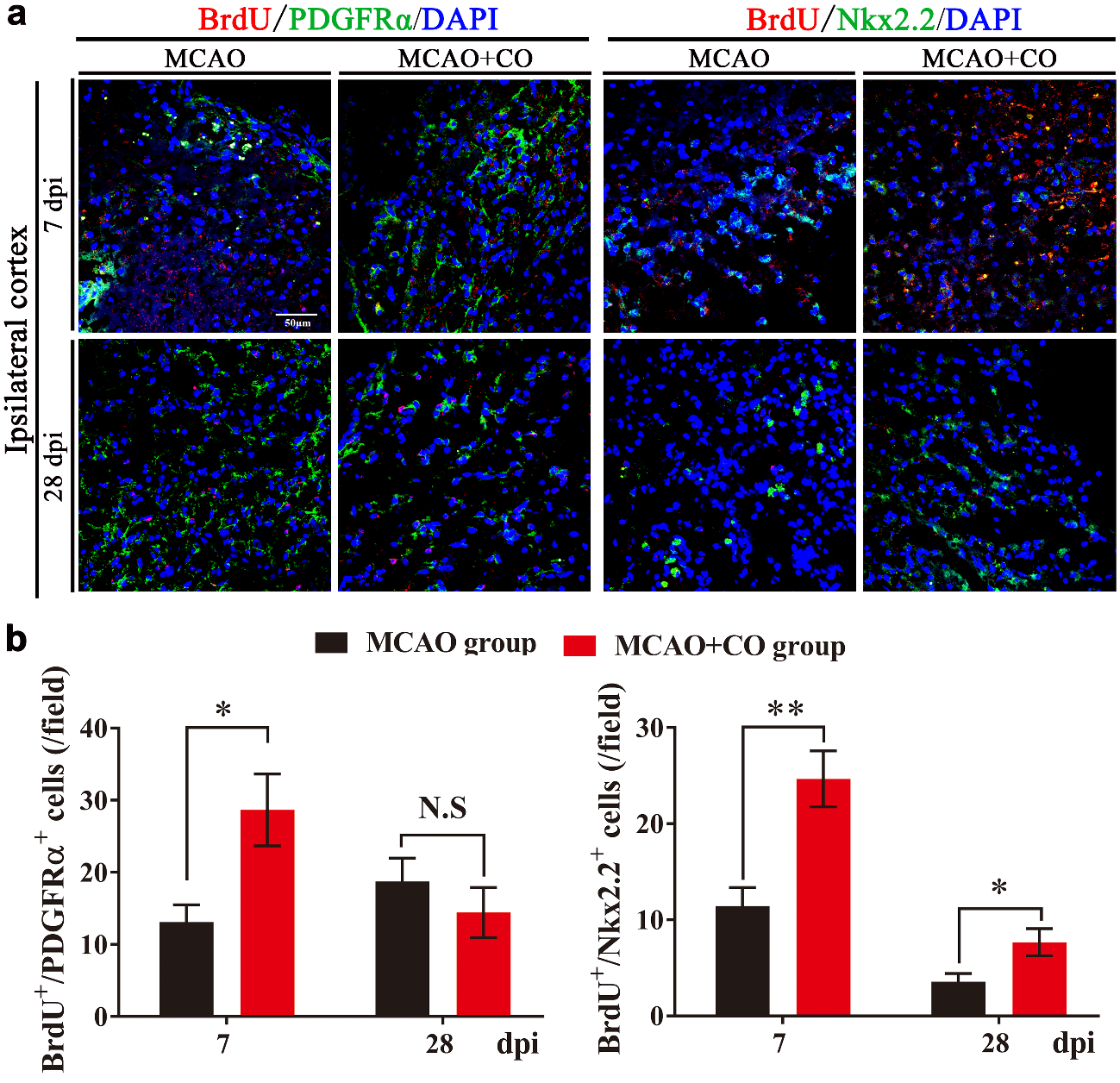


**Supplementary Figure 4. COs transplantation promotes oligodendrogenesis in the ipsilateral cortex of rat MCAO model.** (**a**) Representative images of oligodendrogenesis by co-immunostaining of proliferation cells (BrdU, red) with oligodendrocyte-precursor cells (PDGFRα, green) and oligodendrocyte-lineage cells (Nkx2.2, green) at 7- and 28-day post-implantation (dpi) in the ipsilateral cortex of MCAO and COs transplantation groups. DAPI labels nuclei (blue). Scale bar: 50μm. (**b**) Quantitative analysis of oligodendrogenesis by counting BrdU^+^/ PDGFRα^+^ and BrdU^+^/ Nkx2.2^+^ cells per field at 7 and 28 dpi in the ipsilateral cortex. Immuno-stained positive cells in each group were counted with at least five random microscope fields per section in three rats with ten sections per animal. ^*^*P* < 0.05, ^**^*P* < 0.01. N.S, not significant. All data are shown as mean ± SEM.

**
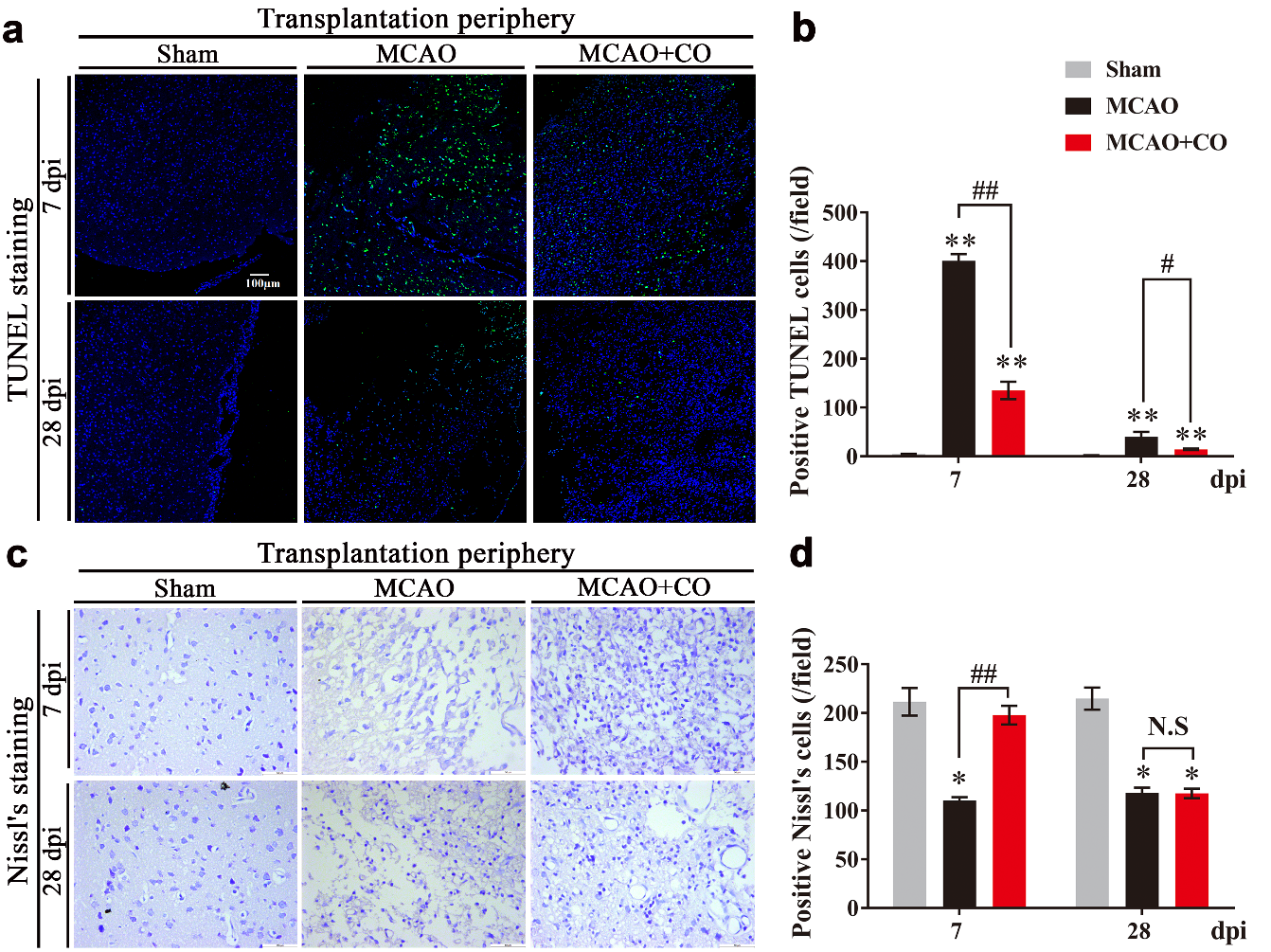
**

**Supplementary Figure 5. COs transplantation decreases neural apoptosis and rescues more survival neurons in the transplantation periphery of ipsilateral cortex of rat MCAO model.** (**a**, **c**) Representative images of cell apoptosis with TUNEL staining and neuronal survival with Nissl’s staining at 7- and 28-day post-implantation (dpi) in the rat transplantation periphery of ipsilateral cortex of Sham, MCAO and COs transplantation groups. All scale bars are as shown. (**b**, **d**) Quantitative analysis of positive TUNEL cells and positive Nissl’s cells per filed in the rat transplantation periphery of ipsilateral cortex. Positive TUNEL cells or Nissl’s cells in each group were counted with at least four random microscope fields per section in three rats with six sections per animal. ^*^*P* < 0.05, ^**^*P* < 0.01 versus Sham group; ^#^*P* < 0.05, ^##^*P* < 0.01 versus MCAO group. N.S, not significant. All data are shown as mean ± SEM.

**
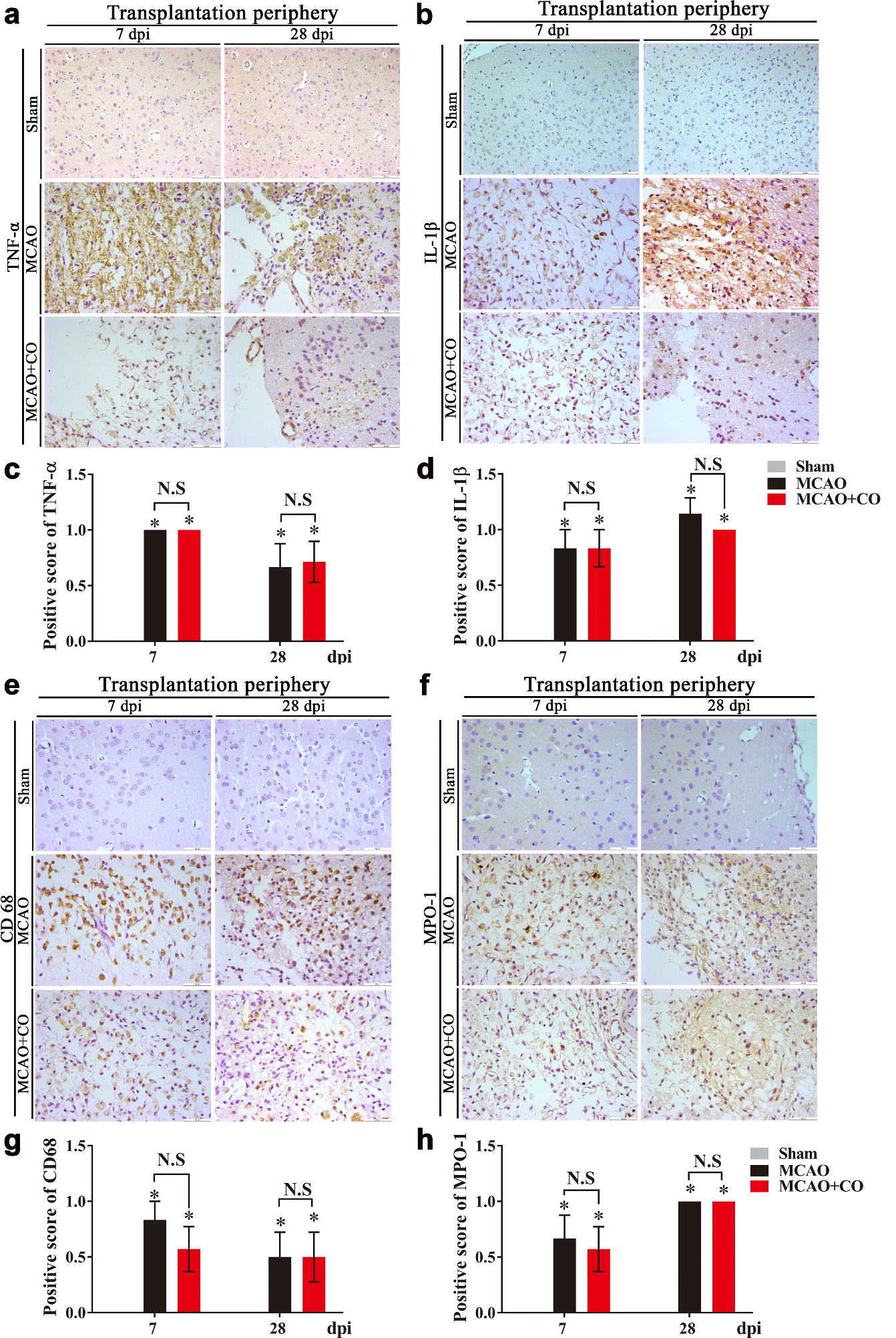
**

**Supplementary Figure 6. COs transplantation has no impact on neuroinflammation in the transplantation periphery of ipsilateral cortex of rat MCAO model.** (**a-b** and **e-f**) Representative images of pro-inflammatory cytokine TNF-α (tumor necrosis factor α) and IL-1 β (interleukin-1 β), phagocytosis marker CD68 and infiltrated neutrophils marker MPO-1 at 7- and 28-day post-implantation (dpi) by immunostaining in the rat transplantation periphery of ipsilateral cortex of Sham, MCAO and COs transplantation groups. All scale bars are as shown. (**c-d** and **g-h**) Quantitative analysis of positive score of TNF-α, IL-1 β, CD68 and MPO-1 expression per field in the rat transplantation periphery of ipsilateral cortex. Positive score of TNF-α, IL-1 β, CD68 and MPO-1 expression in each group were counted with at least four random microscope fields per section in three rats with six sections per animal. ^*^*P* < 0.05 versus Sham groups. N.S, not significant. All data are shown as mean ± SEM.

**
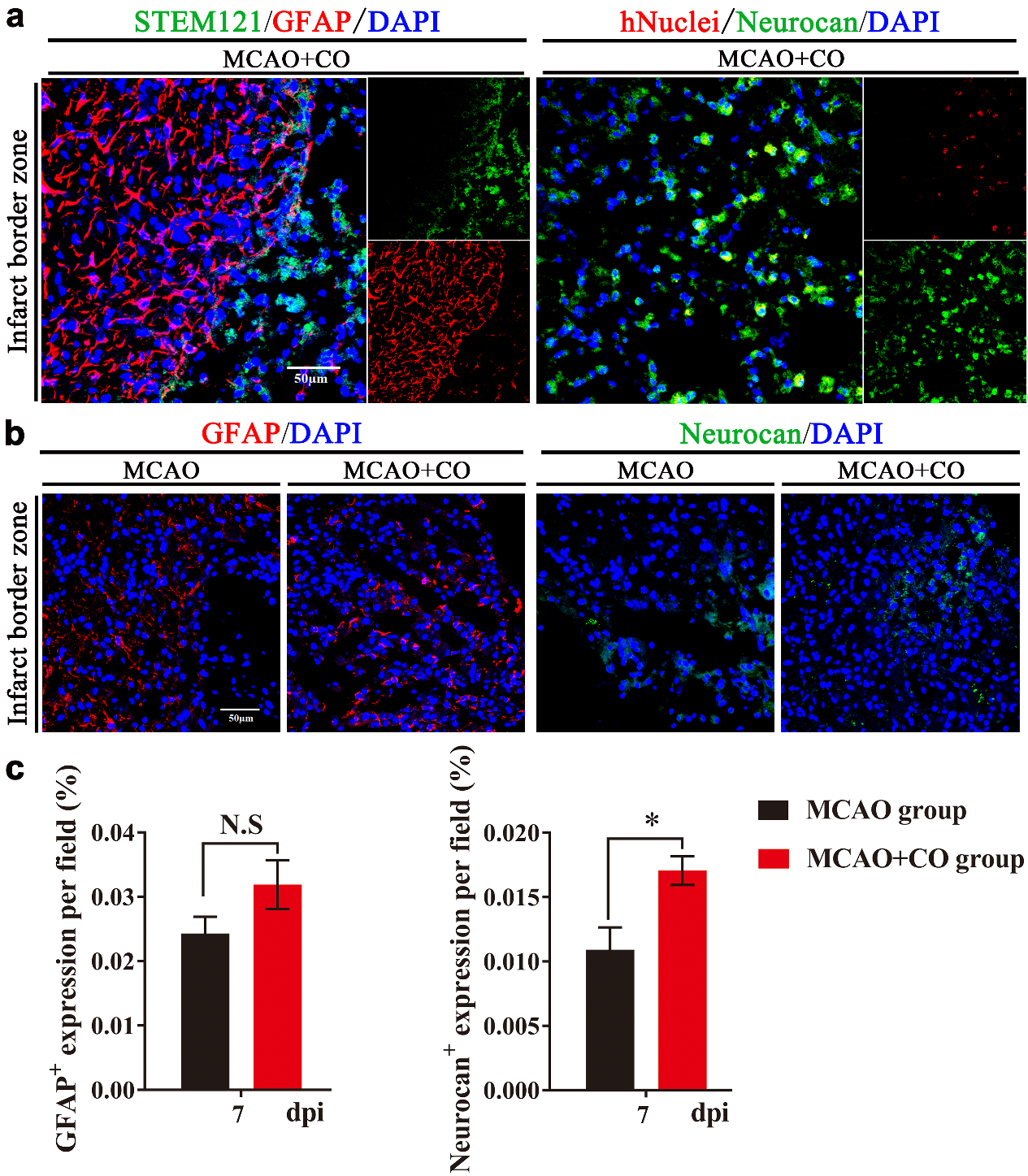
**

**Supplementary Figure 7.** **Transplanted COs take part in the formation of glia scar in the infarct border zone of rat MCAO model.** (**a**) Representative images of *in-vivo* differentiated COs by immunostaining of human cytoplasmic marker STEM121 (green) and astrocytes GFAP (red), human nuclear marker hNuclei (red) and Neurocan (green) at 7-day post-implantation (dpi) in the infarct border zone of COs transplantation group. Scale bar: 50μm. (**b**) Representative images of GFAP and Neurocan expression at 7 dpi in the infarct border zone of MCAO and COs transplantation groups. DAPI labels nuclei (blue). (**c**) Quantitative area ratio of GFAP^+^ expression and Neurocan^+^ expression per field at 7 dpi. The quantitative analysis in each group were counted with at least five random microscope fields per section in three rats with ten sections per animal. ^*^*P* < 0.05. N.S, not significant. All data are shown as mean ± SEM.

**Supplementary Table 1. Antibodies used in this study.**

| **Antibodies** | **Expression** | **Host** | **Reactivity** | **Source/Cat.NO.** | **Dilution** |
| --- | --- | --- | --- | --- | --- |
| SOX 2 | Neural progenitor cell | Rb | H, R | Abcam (ab97959) | 1:250 (ICC/IF) |
| Nestin | Neural stem cell | M | H, R | Abcam (ab6142) | 1:200 (ICC/IF) |
| Tuj1 | Neuron | Rb | H, R | CST (5568s) | 1:200 (ICC/IF) |
| Tuj-1 | Neuron | M | H, R | Abcam (ab78078) | 1:200 (ICC/IF) |
| NeuN | Mature neuron | Rb | H, R | Abcam (ab177487) | 1:300 (ICC/IF) |
| DCX (Doublecortin) | Newborn neuron | Rb | H, R | CST (4604S) | 1:400 (ICC/IF) |
| GFAP | Astrocyte | Rb | H, R | Abcam (ab7260) | 1:300 (ICC/IF) |
| BrdU | Proliferation cell | S | / | Abcam (ab1893) | 1:200 (ICC/IF) |
| Foxg1 | Forebrain | Rb | H, R | Abcam (ab18259) | 1:250 (ICC/IF) |
| TTR | Choroid plexus | S | H, R | Abcam (ab9015) | 1:250 (ICC/IF) |
| Chat | Choline acetyltransferase enzyme | Rb | H, R | Abcam (ab181023) | 1:250 (ICC/IF) |
| Olig2 | Oligodendrocyte and motor progenitor | Rb | H, R | Abcam (ab136253) | 1:250 (ICC/IF) |
| HB9 | Motor neuron | Rb | H, R | Abcam (ab221884) | 1:250 (ICC/IF) |
| TBR1 | Pre-plate/deep-layer neuron | M | H, R | Santa Cruze (SC-376258) | 1:250 (ICC/IF) |
| SATB2 | Surface-layer neuron | M | H, R | Santa Cruze (SC-81376) | 1:250 (ICC/IF) |
| vGlut1 | Glutamatergic neuron | M | H, R | Santa Cruze (sc-377425) | 1:250 (ICC/IF) |
| Synaptophysin | Presynaptic marker | Rb | H, R | Abcam (ab14692) | 1:200 (ICC/IF) |
| PSD 95 | Postsynaptic marker | Rb | R | Abcam (ab18258) | 1:200 (ICC/IF) |
| STEM121 | Human cytoplasm | M | H | Takara (Y40410) | 1:200 (ICC/IF) |
| hNuclei | Human nuclear | Rb | H | Abcam (ab108595) | 1:250 (ICC/IF) |
| CD31 | Endothelial cell | Rb | R | Abcam (ab222783) | 1:100 (ICC/IF) |
| TNFα | Tumor necrosis factor α | Rb | R | Abcam (ab6671) | 1:200 (IHC) |
| IL-1β | Interleukin – 1 β | Rb | R | Abcam (ab9787) | 1:200 (IHC) |
| CD68 | Phagocytosis | Rb | H, R | Abcam (ab125212) | 1:200 (IHC) |
| MPO-1 | Infiltrated neutrophils | Rb | H, R | Abcam (ab9535) | 1:200 (IHC) |

M: mouse; R: rat; Rb: rabbit; S: sheep; H: human. ICC: immunocytochemistry; IF: immunofluorescence; IHC: immunohistochemistry.
